# Phase‐Separation of YAP Mediates AJUBA Super Enhancer Activation to Promote Aberrant Mitosis in Breast Cancer

**DOI:** 10.1002/advs.202409341

**Published:** 2025-12-08

**Authors:** Rui Zhang, Qingwen Huang, Zhuo Chen, Weijian Meng, Hongliang Dong, Zhihong Qi, Liang Liu, Jie Shen, Daxing Xie

**Affiliations:** ^1^ Department of GI Surgery Tongji Hospital Tongji Medical College Huazhong University of Science and Technology Wuhan 430030 China; ^2^ Molecular Medicine Center Tongji Hospital Tongji Medical College Huazhong University of Science and Technology Wuhan 430030 China

**Keywords:** aberrant mitosis, AJUBA, breast cancer, super enhancers, YAP

## Abstract

Aberrant mitosis is a hallmark of cancer, which drives chromosomal instability, gene dysregulation, tumor heterogeneity, immune evasion, and therapy resistance. In this study, it is observed that dysregulation of YAP signaling can cause supernumerary centrosome clustering, thereby triggering pseudo‐bipolar/multipolar diversion during anaphase in breast cancer. Mechanistically, the YAP can accumulate and hyperactivate the super‐enhancer of the spindle assembly checkpoint, *AJUBA*, in a phase‐separation‐dependent manner, thus leading to aberrant mitosis. These findings reveal a crucial biological role of YAP‐mediated super enhancer activation and provide new insights into aneuploidy formation in breast cancer.

## Graphical abstract

YAP undergoes phase separation to activate the *AJUBA* super‐enhancer, driving aberrant mitosis through spindle checkpoint dysregulation in breast cancer. This study uncovers a mechanistic link between YAP condensates, super‐enhancer activation, and aneuploidy formation.

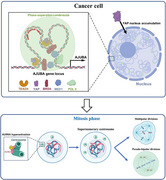

## 1 Introduction

Aneuploidy, characterized by numerical alterations in chromosomes, is a frequent feature of various tumors.^[^ ^1^ ^]^ While traditionally viewed as a consequence of genomic instability, growing evidence suggests that aneuploidy could also play important and active roles in cancer initiation, progression, immune evasion, and therapy resistance.^[^ ^2^ ^]^ One of the major contributors to aneuploidy in cancer cells is centrosome amplification and subsequent aberrant mitosis, which is frequently associated with chromosomal mis‐segregation and poor clinical outcomes.^[^ ^3^, ^4^, ^5^ ^]^ In normal cells, centrosome segregation is tightly regulated to establish two distinct mitotic poles and form a bipolar spindle.^[^ ^6^, ^7^, ^8^ ^]^ In contrast, tumor cells often harbor supernumerary centrosomes, leading to mitotic defects and aneuploidy.^[^ ^9^ ^]^ Given the critical role of centrosome amplification in tumorigenesis, elucidating the oncogenic signaling pathways that drive this process is essential for the development of targeted cancer therapies.

Recent studies have revealed a strong association between Hippo signaling and chromosomal instability (CIN).^[^ ^10^, ^11^ ^]^ Yes‐associated protein (YAP), along with its paralog, transcriptional co‐activator with PDZ‐binding motif (TAZ), functions as a nuclear transcriptional coactivator that regulates organ growth and tissue homeostasis, and has been closely related to tumor aggressiveness and poor prognosis across multiple cancer types.^[^ ^12^, ^13^, ^14^, ^15^ ^]^ Previous reports have shown that YAP promotes aberrant mitotic progression by upregulating the expression of multiple CIN‐associated genes, including *FOXM1*,^[^ ^16^ ^]^ *CDK1*,^[^ ^17^ ^]^ and *SKP2*.^[^ ^18^ ^]^ Despite these findings, the precise transcriptional mechanisms by which YAP drives mitotic defects remain uncertain.

In 2015, Zanconato et al. reported that the YAP/TAZ/TEAD/AP‐1 complex could synergistically regulate target genes that are directly involved in S‐phase entry and mitosis; this control occurred almost exclusively from enhancers that activate target promoters through chromatin looping.^[^ ^19^ ^]^ Further research has reported that YAP/TAZ‐bound enhancers play a key role in the cancer cell state and present active chromatin profiles across diverse human tumors.^[^ ^20^ ^]^ Among these, super‐enhancers (SEs)—clusters of densely occupied enhancers with strong transcriptional activity—have emerged as key regulators of cell fate and tumor progression, and have been reported to mediate YAP/TAZ‐regulated downstream biological processes in various cell types.^[^ ^21^, ^22^, ^23^, ^24^ ^]^ However, how YAP activates SEs to drive mitotic dysregulation remains poorly understood. In particular, whether biophysical mechanisms, such as liquid–liquid phase separation (LLPS), orchestrate SEs activation by YAP has yet to be elucidated.

In this study, we demonstrate that aberrant activation of YAP promotes abnormal mitosis by inducing phosphorylation of AURKA at centrosomes, thereby triggering centrosome amplification and driving aneuploidy in breast cancer cells. Mechanistically, the YAP transcriptional complex accumulates at the super‐enhancer region of *AJUBA*—a key LIM‐domain protein involved in AURKA activation—via LLPS. This phase‐separated condensates facilitate enhancer‐promoter looping, leading to robust transcriptional activation of *AJUBA*. Disruption of the YAP‐SE‐AJUBA axis markedly reduces aberrant mitosis, highlighting a critical pathway linking YAP activity to mitotic fidelity. These findings provide novel mechanistic insight into YAP‐driven genomic instability, and uncover a previously unrecognized mode of Hippo pathway‐mediated transcriptional activation through phase‐separated super‐enhancer complexes. Based on these results, we propose that YAP activation status may serve as a potential biomarker to guide the clinical use of microtubule‐targeting agents in breast cancer therapy.

## 2 Results

### 2.1 YAP‐TEAD Signaling Induces Aberrant Mitosis and Aneuploidy in Breast Cancer

**Figure 1:**
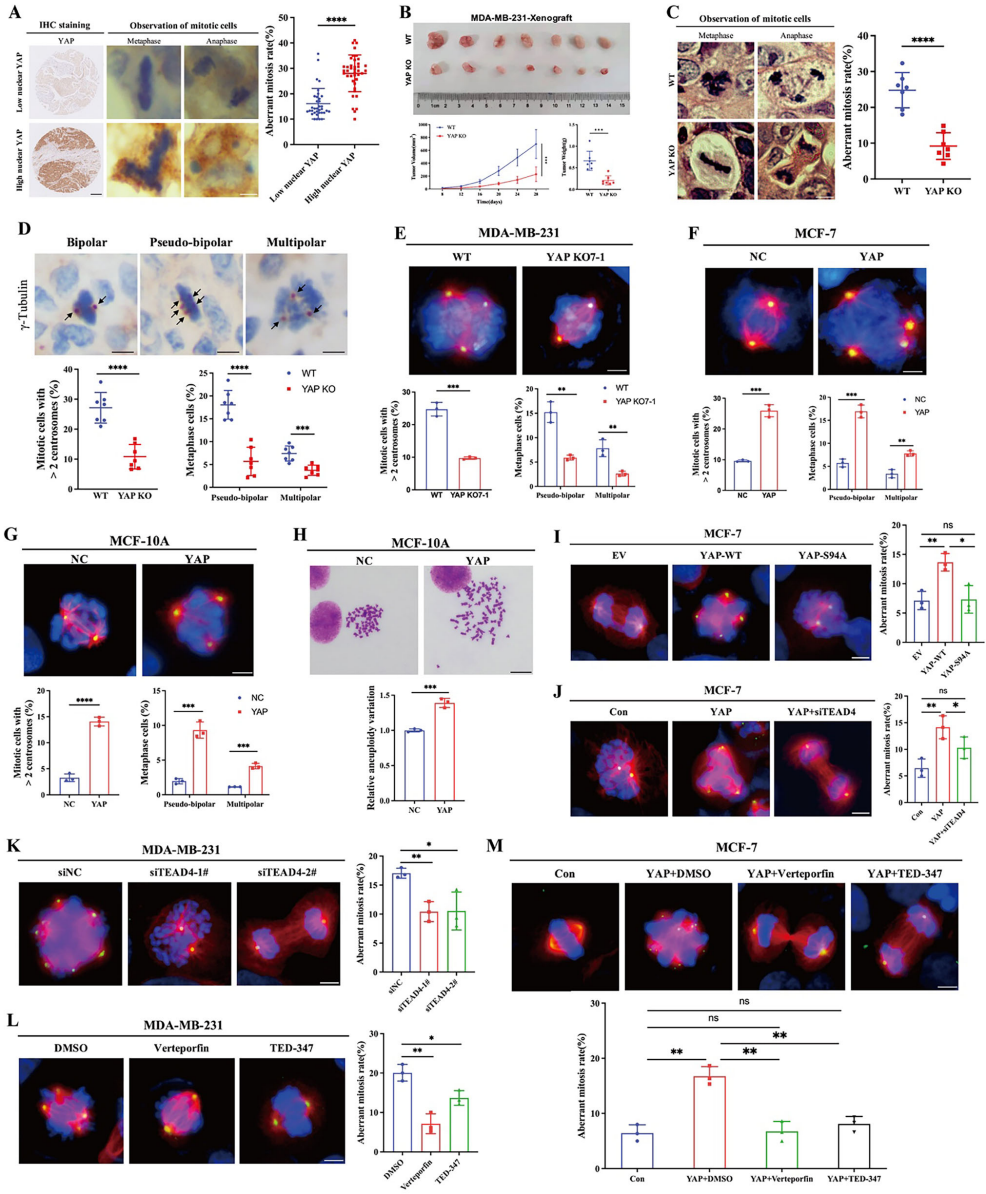
YAP–TEAD signaling induces aberrant mitosis and aneuploidy in breast cancer. A) Representative images of YAP immunohistochemistry staining (scale bar: 250 µm) and aberrant mitotic cells (scale bar: 4 µm) from human triple‐negative breast cancer tissue microarray (*n* = 75). The scatterplot showed the mean percentage ± SD of aberrant mitosis rate. low nuclear YAP, nuclear YAP low expression group (*n* = 35); high nuclear YAP, nuclear YAP high expression group (*n* = 40); ****p < 0.001. B) BALB/c nude mice (*n* = 7) were orthotopically implanted with MDA‐MB‐231 wild‐type cells (WT) or YAP knocking out cells (YAP‐KO). YAP knocking out cells were the mixtures of two YAP‐KO clones (YAP‐KO‐6‐2 and YAP‐KO‐7‐1). Tumor growth curve and tumor weights were measured as indicated. Data were presented as mean ± SD. ***p < 0.001. C) Mitotic cells of xenograft tumors from (B) were observed via H&E staining, and representative images were shown (scale bar: 4 µm). The scatterplot showed the mean percentage ± SD of aberrant mitosis rate. ***p < 0.001. D) Xenograft tumors form (B) were immunohistochemistry stained with γ‐tubulin. Representative images of pseudo‐bipolar, multipolar mitosis and centrosome numbers were shown (scale bar: 4 µm). Arrows pointed to the centrosomes. For each sample, 60–80 metaphase cells were counted. Data were presented as mean percentage ± SD in scatterplots. ***p < 0.001; ****p < 0.001. E–G) Aberrant mitosis was observed by immunofluorescence in indicated stable MDA‐MB‐231, MCF‐7, and MCF‐10A cells, respectively. β‐Tubulin was stained with Alexa Fluor 555 (red), γ‐Tubulin was stained with Dylight 488 (green), and Nuclei were stained with DAPI (blue). Representative images were shown (scale bar: 10 µm). For each experiment, 60–80 mitotic cells were counted, and three independent experiments were performed. Data were presented as mean percentage ± SD in histograms. **p < 0.01; ***p < 0.001; ****p < 0.001. E) wild‐type (WT) and YAP knocking out (YAP KO7‐1) in MDA‐MB‐231 cells. F) negative control (NC) and YAP overexpression (YAP) in MCF‐7 cells. G) negative control (NC) and YAP overexpression (YAP) in MCF‐10A cells. H) Representative images of chromosome metaphase spreading in stable MCF‐10A NC and YAP cells, scale bar: 5 µm. For each experiment, 100–120 cells were counted to quantify the relative aneuploidy variation, and experiments were repeated in triplicates. Data were presented as mean percentage ± SD in histograms. ***p < 0.001. I–M) Aberrant mitosis was observed by immunofluorescence in the indicated MDA‐MB‐231 and MCF‐7 cells. β‐Tubulin was stained with Alexa Fluor 555 (red), γ‐Tubulin was stained with Dylight 488 (green), and Nuclei were stained with DAPI (blue). Representative images were shown and scale bar: 10 µm. For each experiment, 60–80 mitotic cells were counted, and three independent experiments were performed. Histograms showed the mean percentage ± SD of aberrant mitosis rate. ns, not statistically significant; *p < 0.05; **p < 0.01. I) MCF‐7 cells transfected with empty vector (EV), wild‐type YAP (YAP‐WT), or YAP‐S94A mutant (YAP‐S94A). J) MCF‐7 cells with control (Con), YAP overexpression (YAP), or YAP overexpression plus *TEAD4* knocking down (YAP+siTEAD4). K) MDA‐MB‐231 transfected with scramble siRNA (siNC) or *TEAD4* siRNA (siTEAD4‐1#, TEAD4‐2#). L) MDA‐MB‐231 cells were treated with DMSO, Verteporfin (at a dose of 1 µM), or TED‐347 (at a dose of 10 µM) for 16 h, respectively. M) MCF‐7 YAP overexpression (YAP) and control (Con) stable cells were treated with DMSO, Verteporfin (at a dose of 1 µM), or TED‐347 (at a dose of 10 µM) for 16 h, respectively.

To investigate the correlation between YAP expression and aberrant mitoses in clinical breast cancer, we first performed immunohistochemical (IHC) staining of YAP on a tissue microarray comprising 75 primary breast cancer specimens. Based on IHC scores, samples were stratified into low nuclear YAP (*n* = 35) and high nuclear YAP (*n* = 40) expression groups. Quantification of mitotic cells revealed that tumors with high nuclear YAP expression exhibited a significantly higher frequency of aberrant mitoses (**Figure** **1**A).

To determine whether YAP directly contributes to aberrant mitoses, we selected two commonly used breast cancer cell lines based on their endogenous YAP levels: MDA‐MB‐231 (high YAP expression) and MCF‐7 (low YAP expression), as previously reported.^[^ ^25^ ^]^ In MDA‐MB‐231 cells, endogenous YAP was knocked out using CRISPR/Cas9 (Figure S1A, Supporting Information). Based on knockout efficiency, the KO 7‐1 clone was selected for subsequent experiments, and orthotopic xenografts were then established in female NOD/SCID mice. Four weeks post‐implantation, tumors were harvested for analysis. YAP knockout led to a marked reduction in tumor growth and cell proliferation compared with control cells (Figure 1B; Figure S1B, Supporting Information). Consistently, histological analysis revealed a significant reduction in the proportion of mitotic abnormalities in YAP‐deficient xenograft tumors (Figure 1C). Furthermore, YAP knockout markedly decreased the frequency of spontaneous pseudo‐bipolar and multipolar divisions, with mitotic cells exhibiting a lower incidence of abnormal centrosome amplification (Figure 1D), thereby implicating YAP as a critical regulator of mitotic aberrations and centrosome dynamics.

To further investigate the role of YAP in aberrant mitosis and supernumerary centrosome clustering, we performed immunofluorescence staining with antibodies against γ‐tubulin (a centrosome marker) and β‐tubulin (a spindle marker) to visualize abnormal mitotic configurations, including pseudo‐bipolar and multipolar spindles (Figure S1C, Supporting Information). In MDA‐MB‐231 cells, YAP knockout significantly decreased the proportion of cells undergoing spontaneous pseudo‐bipolar and multipolar mitoses, while concurrently reducing the percentage of mitotic cells with abnormal centrosome amplification (Figure 1E). Conversely, YAP overexpression in MCF‐7 cells (Figure S1D, Supporting Information) led to a marked increase in aberrant mitosis and supernumerary centrosome clustering (Figure 1F). Similarly, enforced expression of YAP in the non‐tumorigenic breast epithelial cell line MCF‐10A (Figure S1E, Supporting Information) induced prominent pseudo‐bipolar and multipolar mitoses and centrosome amplification (Figure 1G). To determine whether YAP‐induced aberrant mitosis is associated with specific molecular subtypes of breast cancer, we also conducted experiments in BT‐549 cells (a triple‐negative breast cancer cell line with low endogenous YAP expression) and T‐47D cells (an ER⁺ cell line with high endogenous YAP expression). The findings were consistent with those observed in MCF‐7 and MDA‐MB‐231 cells (Figure S1F–I, Supporting Information), supporting the notion that YAP regulates aneuploidy irrespective of breast cancer subtype.

Aberrant mitosis and cytokinesis failure are considered the most common routes leading to aneuploidy in cancer cells.^[^ ^26^, ^27^, ^28^ ^]^ Further karyotype analysis revealed that YAP expression was positively correlated with high modal chromosomal number in both MDA‐MB‐231 and MCF‐7 cells (Figure S1J,K, Supporting Information). Moreover, overexpression of YAP in MCF‐10A cells resulted in the accumulation of aneuploid cells, indicating that YAP‐induced chromosomal instability is not limited to cancer cells (Figure 1H).

To determine whether this phenotype is specific to YAP or also applies to its paralog, we examined the effect of TAZ overexpression. Consistent with YAP, TAZ also promoted aberrant mitoses in breast cancer cells (Figure S2A–D, Supporting Information), suggesting that both YAP and TAZ could contribute to mitotic defects and chromosomal instability. Next, we investigated whether TEAD, the key downstream transcription factor of YAP/TAZ, is required for YAP/TAZ‐induced mitotic abnormalities. To this end, we transfected MCF‐7 cells with either wild‐type YAP or a TEAD‐binding‐deficient mutant (YAP‐S94A) plasmid (Figure S2E, Supporting Information). As expected, wild‐type YAP significantly increased the incidence of aberrant mitoses, whereas this effect was abolished in cells transfected with the YAP‐S94A mutant instead (Figure 1I), indicating that TEAD binding is essential for YAP‐driven mitotic defects.

Among the *TEAD* family members, *TEAD4* is highly expressed in breast cancer and has been reported to correlate with poor prognosis.^[^ ^25^ ^]^ To determine its functional role, we transiently knocked down *TEAD4* in YAP‐overexpressed MCF‐7 cells (Figure S2F, Supporting Information) and MDA‐MB‐231 cells (Figure S2G, Supporting Information). TEAD4 knockdown markedly reversed the aberrant mitotic phenotypes induced by YAP/TAZ overexpression (Figure 1J,K). Furthermore, pharmacologic inhibition of YAP‐TEAD interaction using small‐molecule inhibitors (verteporfin and TED‐347) (Figure S2H,I, Supporting Information) significantly reduced the proportion of cells displaying aberrant mitoses and supernumerary centrosome clustering (Figure 1L,M). These findings collectively support that TEAD‐dependent transcriptional activity is essential for YAP/TAZ‐induced mitotic aberrations and centrosome amplification.

### 2.2 YAP Activates the Centrosome‐Localized Mitotic Kinase AURKA

**Figure 2:**
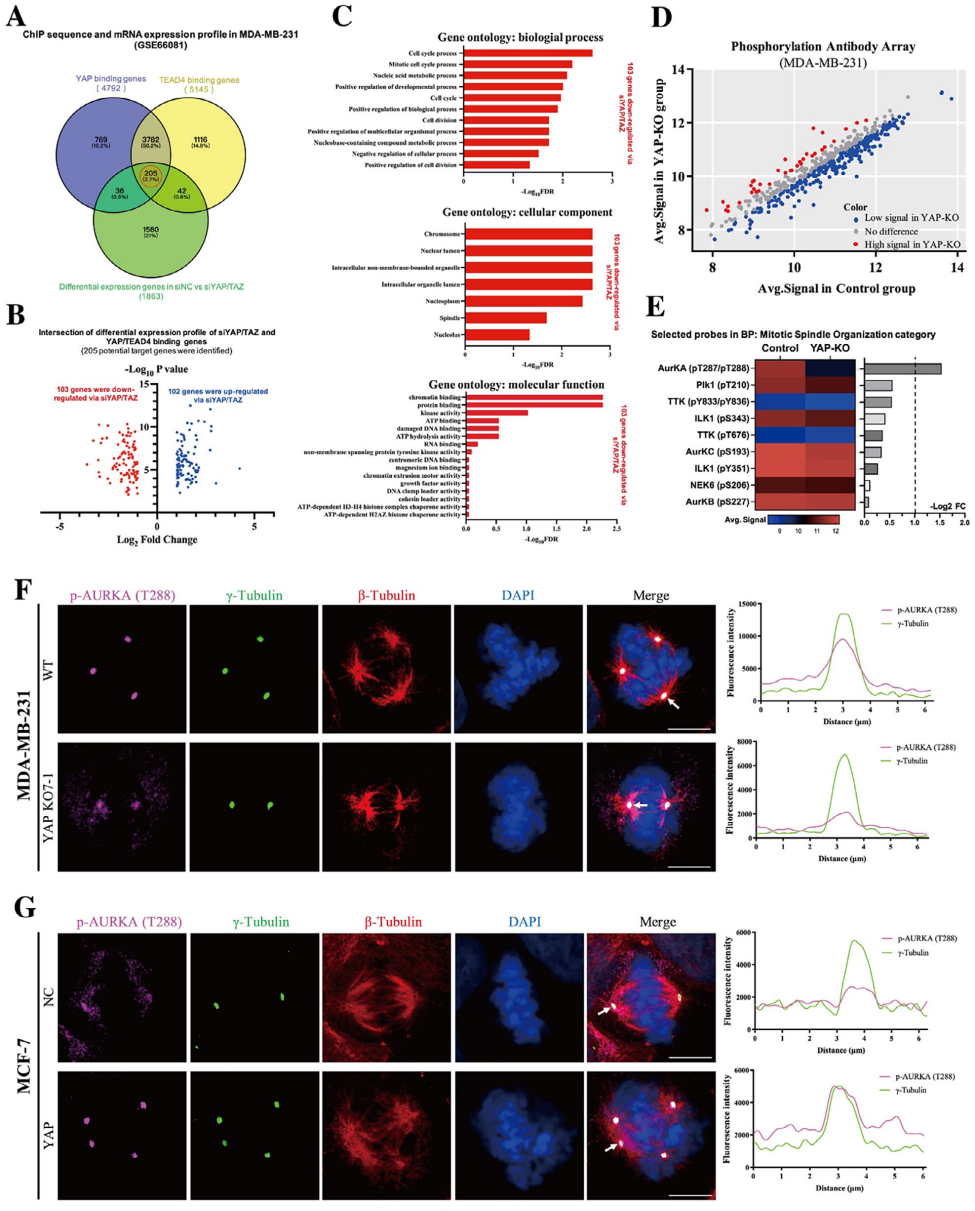
YAP activates the centrosome‐localized mitotic kinase AURKA. A) A Venn diagram indicated the intersection between YAP/TEAD binding genes and the differential expression genes of siNC versus siYAP/TAZ in MDA‐MB‐231 cells. Data were obtained from GSE66081 and GSE66082. 205 intersected genes (central red circle) were collected for further analysis. B) The volcano diagram showed the differential expression level of intersected genes from (A). 103 genes (red dots) were downregulated, while 102 genes (blue dots) were upregulated via siYAP/TAZ. C) Gene ontology enrichment of the 103 downregulated genes indicated in (B). Biological processes, cellular components, and molecular functions involved were presented. Bar plots represent ‐Log_10_ false discovery rate (FDR). D) RayBio Label‐Based (L‐Series) Human Phosphorylation Screening Array kit was used to investigate protein phosphorylation level in MDA‐MB‐231 WT and YAO‐KO cells. The scatterplot showed the average protein phosphorylation signal intensity in two groups, red dots: high signal in YAP‐KO; blue dots: low signal in YAP‐KO; gray dots: no difference. E) Quantification of differential protein phosphorylation signal intensity within the mitotic spindle organization category from (D). Bar plots and a heatmap showed the Log_2_ fold change values (Log_2_FC) of average signal intensity in two groups. F,G) Representative images showed AURKA activation at the centrosome via immunofluorescence in MDA‐MB‐231 WT and YAP‐KO (F), or MCF‐7 NC and YAP (G) cells, respectively. p‐AURKA (T288) was stained with Alexa Fluor 647 (violet), γ‐Tubulin was stained with Dylight 488 (green), β‐Tubulin was stained with Alexa Fluor 555 (red), and Nuclei were stained with DAPI (blue). Scale bar: 10 µm. Line plot analyzed the colocalization and fluorescence intensity of p‐AURKA (T288) and γ‐Tubulin.

To elucidate the mechanism by which YAP promotes aberrant mitosis and aneuploidy, we reanalyzed publicly available chromatin immunoprecipitation sequencing (ChIP‐seq) data for YAP/TEAD4 binding (GSE66081) alongside transcriptomic profiles of *YAP/TAZ* knockdown in MDA‐MB‐231 cells (GSE66082).^[^ ^19^ ^]^ By integrating ChIP‐seq and mRNA expression data, we identified 3987 genes co‐occupied by YAP and TEAD4, of which 205 were differentially expressed upon *YAP*/*TAZ* knockdown (Figure **2**A). Among these, 102 genes were downregulated and 103 were upregulated following *YAP/TAZ* silencing (Figure 2B), indicating that they represent potential direct transcriptional targets of YAP/TEAD. Gene Ontology (GO) analysis revealed that the YAP/TEAD‐downregulated genes were significantly enriched in biological processes related to the “mitotic cell cycle” and “cell division”, suggesting a potential role for YAP in mitotic regulation (Figure 2C). Furthermore, subcellular component and molecular function analyses demonstrated that these YAP‐regulated genes were enriched in terms associated with the nucleus, spindle apparatus, and kinase activity, pointing to a potential mechanism by which YAP could influence mitotic progression and centrosome behavior (Figure 2C).

Mitotic regulation involves the coordinated action of multiple cell cycle‐related kinases. To determine whether YAP influences the activity of these mitotic kinases, we performed a phospho‐protein screening microarray in MDA‐MB‐231 cells with or without *YAP* knockout. The results revealed a global reduction in phosphorylation levels of several cell cycle‐related proteins upon *YAP* deletion (Figure 2D,E). Among the downregulated phospho‐proteins, nine kinases were directly implicated in mitotic spindle organization, suggesting their potential involvement in YAP‐mediated supernumerary centrosome clustering and aberrant mitosis. Notably, the phosphorylation level of AURKA at threonine 287/288 (pT287/pT288)—a critical modification required for its activation—was the most significantly reduced among these mitotic regulators (Figure 2E). Given the established role of AURKA in centrosome maturation and mitotic spindle assembly,^[^ ^29^, ^30^ ^]^ we selected AURKA as a key candidate for further functional investigation. Immunofluorescence staining further demonstrated that YAP regulated the activation of AURKA specifically at the centrosome during mitosis (Figure 2F,G). Moreover, pharmacological inhibition of AURKA activity using the selective inhibitor alisertib (MLN8237) effectively suppressed YAP‐induced AURKA phosphorylation at threonine 288 and significantly reduced the incidence of aberrant mitoses (Figure S3A,B, Supporting Information). Together, these findings suggest that YAP promotes aberrant mitosis, at least in part, by activating centrosomal AURKA.

### 2.3 YAP Activates AURKA through Transcriptional Upregulation of *AJUBA*

**Figure 3:**
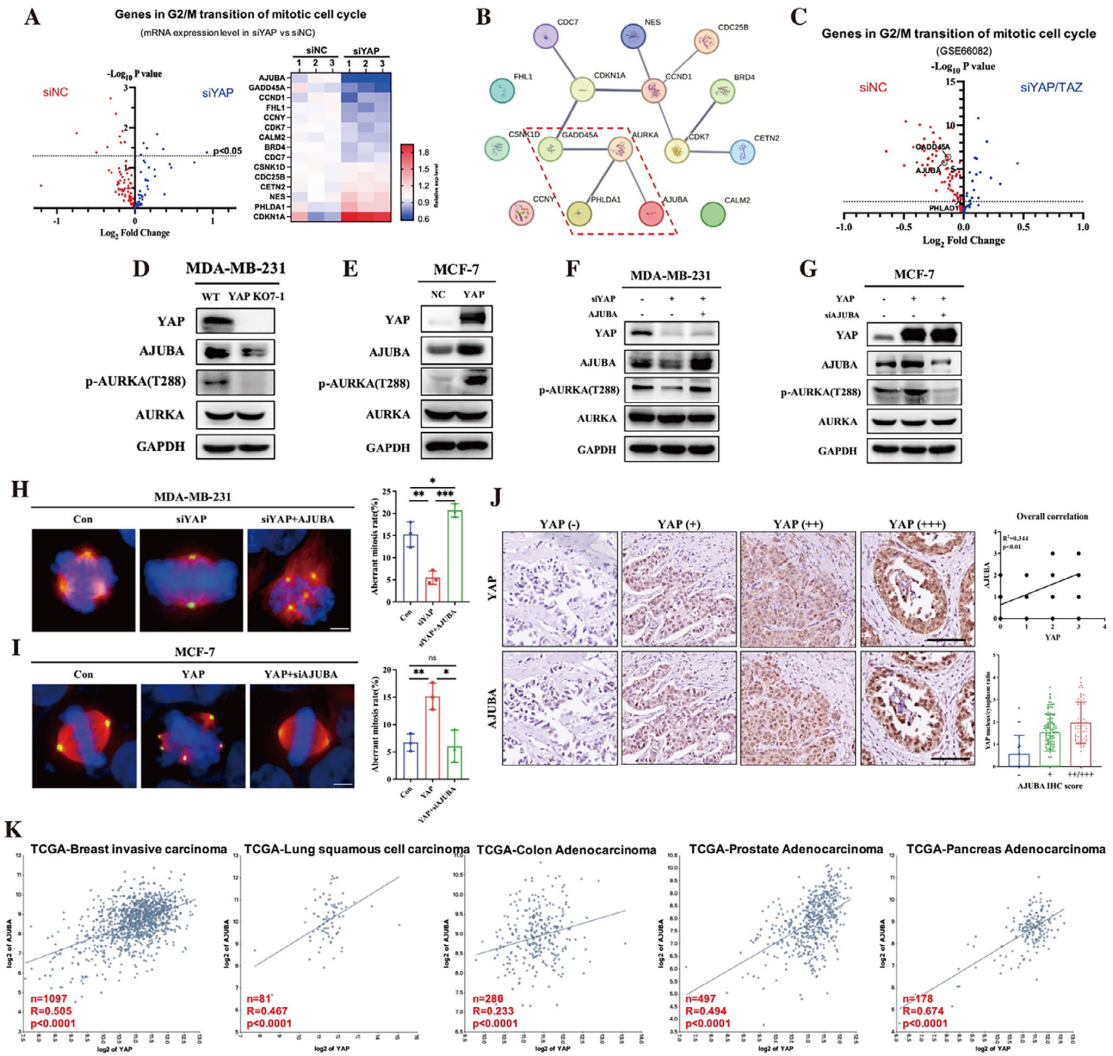
YAP activates AURKA through transcriptional upregulation of *AJUBA*. A) Volcano diagram (left) indicated the differential expression genes within G2/M transition of the mitotic cell cycle of siYAP versus siNC in MDA‐MB‐231 cells. Red dots were downregulated, and blue dots were upregulated via siYAP. Heatmap (right) showed the expression profiles of genes exhibited statistically significant differences (*p* < 0.05) between siYAP and siNC. B) Potential protein–protein interactions (PPI) between AURKA and its regulators were analyzed and visualized via the STRING database. C) Volcano diagram indicated the differential expression genes within G2/M transition of the mitotic cell cycle of siNC versus siYAP/TAZ in MDA‐MB‐231 cells. Data were obtained from GSE66082. Red dots were downregulated, and blue dots were upregulated via siYAP/TAZ. D) Cell lysates from MDA‐MB‐231 WT and YAP KO7‐1 cells were collected for immunoblot. Lysates were probed for YAP, AJUBA, AURKA, and p‐AURKA (T288). GAPDH was used as a loading control. E) Cell lysates from MCF‐7 NC and YAP cells were collected for immunoblot. Lysates were probed for YAP, AJUBA, AURKA, and p‐AURKA (T288). GAPDH was used as a loading control. F) Cell lysates from MDA‐MB‐231 cells transfected with *YAP* siRNAs (siYAP) and/or AJUBA overexpressing plasmid (AJUBA) were collected for immunoblot. Empty vector and scramble siRNA (siNC) were used as a negative control. Lysates were probed for YAP, AJUBA, AURKA, and p‐AURKA (T288). GAPDH was used as a loading control. G) Cell lysates from MCF‐7 cells transfected with YAP overexpressing plasmid (YAP) and/or *AJUBA* siRNAs (siAJUBA) were collected for immunoblot. Empty vector and scramble siRNA (siNC) were used as a negative control. Lysates were probed for YAP, AJUBA, AURKA, p‐AURKA (T288), and GAPDH was used as a loading control. H,I) Aberrant mitosis was observed by immunofluorescence from MDA‐MB‐231 (F) and MCF‐7 (G) cells treated as indicated. β‐Tubulin was stained with Alexa Fluor 555 (red), γ‐Tubulin was stained with Dylight 488 (green), and Nuclei were stained with DAPI (blue). Representative images were shown and scale bar: 10 µm. For each experiment, 60–80 mitotic cells were counted, and three independent experiments were performed. Histograms showed the mean percentage ± SD of aberrant mitosis rate. ns, not statistically significant; *p < 0.05; **p < 0.01; ***p < 0.001. J) Representative images of YAP and AJUBA IHC staining from tumors with different YAP expression levels in human pan‐cancer specimen microarray (scale bar: 250 µm). This array contained multiple tumors including breast invasive ductal carcinoma (*n* = 37), lung squamous cell carcinoma (*n *= 39), colorectal adenocarcinoma (*n *= 38), prostate adenocarcinoma (*n *= 38), and pancreas adenocarcinoma (*n *= 38). YAP (−): YAP IHC intensity = 0, *n *= 26; YAP (+): YAP IHC intensity =1, *n* = 95; YAP (++): YAP IHC intensity=2, *n* = 47; and YAP (+++): YAP IHC intensity = 3, *n *= 22. The correlation between YAP and AJUBA IHC staining intensity was evaluated via IHC score and calculated by Pearson analysis, *R* ^2 ^= 0.344 and *p *< 0.01 (right top). The correlation between YAP nucleus/cytoplasm ratio and AJUBA IHC score was presented as mean ± SD in scatterplots (right bottom). K) Validation of correlation between YAP and AJUBA expression levels in the TCGA cancer database corresponding with human pan‐cancer specimen microarray in (J). And the correlation between YAP and AJUBA was calculated by Pearson analysis, *R* ^2^, and *p* were indicated in the Scatterplot.

To investigate how YAP directly promotes AURKA activation, we performed high‐throughput mRNA sequencing in MDA‐MB‐231 cells transiently transfected with *YAP* siRNAs. Among the genes associated with the “G2/M transition of mitotic cell cycle” category, we identified 15 genes with significant differential expression (*p* < 0.05) (Figure **3**A). Protein–protein interaction (PPI) network analysis revealed that GADD45A and AJUBA (both downregulated upon *YAP* knockdown), as well as PHLDA1 (upregulated upon *YAP* knockdown), showed high‐confidence interactions with AURKA (Figure 3B). To further validate these findings, we analyzed gene expression in the previously reported public dataset GSE66082. Consistently, both *AJUBA* and *GADD45A* were significantly downregulated in siYAP/TAZ‐treated cells, while the change in *PHLDA1* expression was not statistically significant (Figure 3C).

GADD45A is a well‐known cellular stress sensor that is activated in response to DNA damage or chromosome mis‐segregation, and promotes DNA repair.^[^ ^31^ ^]^ This finding provides indirect support for the YAP‐induced aneuploidy phenotype observed in our study. In contrast, AJUBA, a LIM domain‐containing protein, acts upstream of AURKA and is known to promote its activation by enhancing phosphorylation at threonine 288, thereby facilitating mitotic spindle assembly.^[^ ^32^, ^33^, ^34^ ^]^ Based on this mechanistic role and its consistent downregulation upon *YAP* silencing, *AJUBA* was selected for further investigation. To determine whether YAP regulates AJUBA expression, we performed western blot analysis in MDA‐MB‐231 and MCF‐7 cells. The results showed that both YAP and TAZ positively regulated AJUBA protein levels (Figure 3D,E; Figure S4A,B, Supporting Information). Furthermore, pharmacologically inhibiting the YAP‐TEAD interaction significantly attenuated YAP‐induced AJUBA expression in both cell lines (Figure S4C–F, Supporting Information), indicating that *AJUBA* is a TEAD‐dependent transcriptional target of YAP/TAZ.

To determine whether YAP‐induced aberrant mitosis is mediated by AJUBA, we conducted both gain‐ and loss‐of‐function experiments in MDA‐MB‐231 and MCF‐7 cells. Specifically, *AJUBA* was knocked down or overexpressed, and the downstream effects on AURKA activity and mitotic behavior were evaluated. Our results demonstrated a positive correlation between AJUBA expression and AURKA phosphorylation at threonine 288 (Figure S4G,H, Supporting Information), as well as the rate of aberrant mitoses (Figure S4I,J, Supporting Information). Importantly, overexpression of *AJUBA* rescued AURKA phosphorylation in *YAP*‐knockdown MDA‐MB‐231 cells (Figure 3F). Conversely, silencing *AJUBA* significantly suppressed AURKA T288 phosphorylation in both MCF‐7 and MCF‐10A cells with ectopic *YAP* expression (Figure 3G; Figure S4K, Supporting Information). Furthermore, *AJUBA* depletion also reversed YAP‐induced mitotic abnormalities across multiple cell types, including MDA‐MB‐231, MCF‐7, and the non‐tumorigenic epithelial cell line MCF‐10A (Figure 3H,I; Figure S4L, Supporting Information). These findings collectively indicate that AJUBA is a critical downstream effector through which YAP activates AURKA and promotes mitotic aberrations.

We further validated the association between YAP and AJUBA expression in clinical cancer specimens. IHC staining of a multi‐cancer tissue array comprising breast invasive ductal carcinoma (*n* = 37), lung squamous cell carcinoma (*n* = 39), colorectal adenocarcinoma (*n* = 38), prostate adenocarcinoma (*n* = 38), and pancreatic adenocarcinoma (*n* = 38) revealed a positive correlation between YAP and AJUBA protein levels across multiple tumor types. Moreover, AJUBA expression was positively correlated with nuclear YAP accumulation (Figure 3J). In addition, analysis of public datasets from The Cancer Genome Atlas (TCGA) using the R2 Genomics Platform confirmed a strong positive correlation between *YAP* and *AJUBA* mRNA expression across multiple cancer types (Figure 3K). These results support the notion that YAP transcriptionally upregulates *AJUBA*, which in turn activates AURKA and promotes mitotic abnormalities, providing a mechanistic link between YAP signaling and chromosomal instability in human cancers.

### 2.4 YAP Promotes *AJUBA* Transcription via Super‐Enhancer Activation

**Figure 4:**
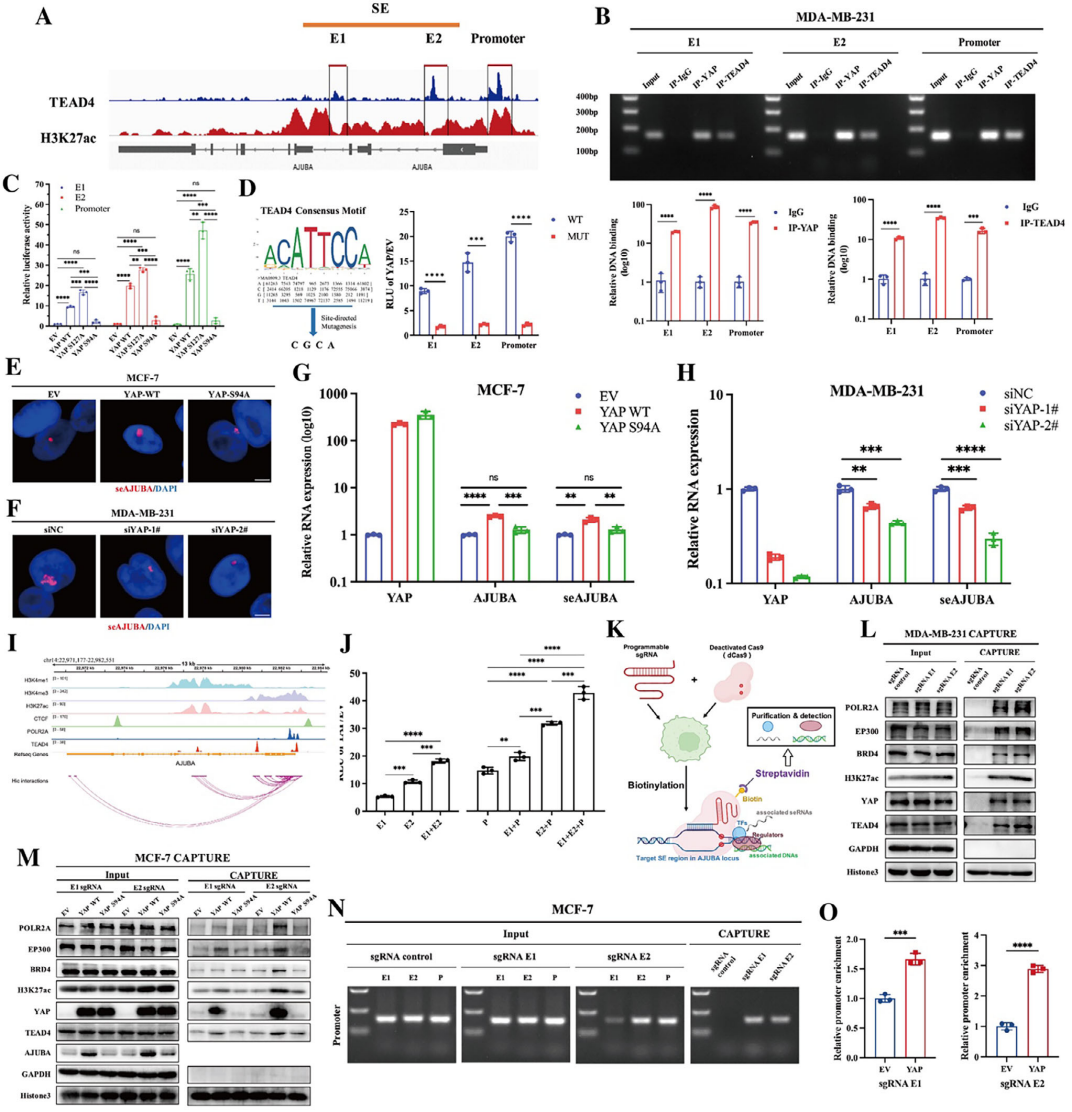
YAP promotes *AJUBA* transcription via super‐enhancer Activation. A) ChIP‐seq track diagram of H3K27ac and TEAD4 on the *AJUBA* locus. Data were obtained from the ENCODE database. SE: Super enhancer; E1: Enhancer 1; E2: Enhancer 2. B) A ChIP assay was performed using YAP or TEAD4 antibodies in MDA‐MB‐231 cells. IgG antibody was used as a negative control. DNA in the immunocomplex was amplified with the primers targeting E1, E2, or *AJUBA* promoter, and measured via agarose gel electrophoresis (upper) or qPCR (lower). ***p < 0.001; ****p < 0.0001. C) A dual Luciferase reporter assay was performed to evaluate the relative transcriptional activity of E1, E2, or *AJUBA* promoter in HEK‐293T cells transfected with empty vector (EV), wild‐type YAP (YAP WT), YAP S127A mutant (YAP S127A), or YAP S94A mutant (YAP S94A), respectively. ns, not statistically significant; **p < 0.01; ***p < 0.001; ****p < 0.0001. D) Left: diagram of site‐directed mutagenesis on TEAD4 motif. The TEAD4 motif sequence was obtained from the JASPAR database. Right: Dual Luciferase report assay was performed on HEK‐293T cells with EV or YAP overexpression. The transcriptional activity of E1, E2, and *AJUBA* promoters with wild‐type or mutated TEAD4 motif was evaluated by the ratio of RLU between cells with YAP and EV transfected. RLU, relative light unit. ***p < 0.001 and ****p < 0.0001. E,F) RNA‐FISH assay was performed to detect *AJUBA* seRNA expression in MCF‐7 cells with EV, YAP‐WT, or YAP‐S94A overexpression (E), and in MDA‐MB‐231 cells transfected with scramble siRNA (siNC) or *YAP* siRNA (siYAP‐1#, siYAP‐2#) (F). The representative images were shown, scale bar: 5 µm. Probes for seRNA were conjugated with Cy3 (red), and the nuclei were stained with DAPI (blue). G,H) qPCR detected the expression level of *AJUBA* seRNA (*seAJUBA*) and mRNA in MCF‐7 EV, YAP‐WT, or YAP‐S94A (G), and in MDA‐MB‐231 siNC, siYAP‐1#, or siYAP‐2# (H). Each experiment was repeated in triplicates, and *GAPDH* was used as an endogenous control. ns, not statistically significant; **p < 0.01; ***p < 0.001; ****p < 0.0001. I) The chromatin‐chromatin interactions between promoter and E1/E2 of the *AJUBA* gene in MCF‐7 cells were analyzed via Hi‐C data from the ENCODE database, and were shown as indicated. H3K4me1, H3K4me3, and H3K27ac were used to mark the super enhancer. POLR2A was used to mark the transcriptional starting site, and CTCF was used to mark the topologically associating domain. TEAD4 binding sites on the *AJUBA* locus in MCF‐7 were also marked in the diagram. J) A Dual luciferase reporter assay was performed on HEK‐293T cells with empty vector (EV) or YAP overexpression. The transcriptional activity of *AJUBA* E1, E2, promoter (P), E1+E2, E1+P, E2+P and E1+E2+P were evaluated. The transcriptional activity was normalized by the ratio of RLU between cells with YAP and EV transfected. RLU, relative light unit. **p < 0.01; ***p < 0.001; ****p < 0.0001. K) Diagram to elucidate the operational principles of the CRISPR affinity purification in situ of regulatory elements (CAPTURE) system. L) CAPTURE assay was performed to evaluate the potential binding proteins on the E1/E2 locus of the *AJUBA* gene in MDA‐MB‐231 cells. Polycistronic sgRNAs targeting E1/E2 were used in the immunoprecipitation; and the protein levels of POLR2A, EP300, BRD4, H3K27ac, YAP, and TEAD4 were examined via western blot. GAPDH and Histone were used as loading controls. M) CAPTURE assay was performed to evaluate the potential binding proteins on the E1/E2 locus of the *AJUBA* gene in MCF‐7 cells with EV, YAP WT, or YAP S94A transfected. Polycistronic sgRNAs targeting E1/E2 were used in the immunoprecipitation; and the protein levels of POLR2A, EP300, BRD4, H3K27ac, YAP, and TEAD4 were examined via western blot. GAPDH and Histone were used as loading controls. N) CAPTURE assay was performed to validate the potential interaction between promoter and E1/E2 locus of *AJUBA* gene in MCF‐7 cells. Polycistronic sgRNAs targeting E1/E2 were used in the immunoprecipitation. The input and affinity‐captured DNA products were amplified using primers targeting the *AJUBA* promoter and analyzed by agarose gel electrophoresis. O) CAPTURE assay was performed to validate the potential interaction between promoter and E1/E2 locus of *AJUBA* gene in MCF‐7 cells transfected with YAP or EV. The affinity‐captured DNA products were analyzed by qPCR using primers targeting the *AJUBA* promoter. ***p < 0.001; ****p < 0.0001.

To elucidate the mechanism by which YAP induces *AJUBA* transcription, we analyzed publicly available ChIP‐seq data for MCF‐7 cells from the ENCODE database. Three TEAD4‐binding peaks were identified within the *AJUBA* genomic locus. One peak was located within the conventional promoter region (−2000 to +50 bp relative to the transcription start site), while the other two peaks (designated E1 and E2) overlapped with H3K27ac‐enriched regions—hallmarks of active enhancers—and were classified as putative super‐enhancers based on rank ordering of super enhancers (ROSE) analysis (**Figure** **4**A).

We next confirmed TEAD4 and YAP binding at the *AJUBA* promoter, E1, and E2 regions in MDA‐MB‐231 cells using chromatin immunoprecipitation (ChIP) assays (Figure 4B). To assess their transcriptional activity, we constructed luciferase reporter vectors containing the *AJUBA* promoter or the E1/E2 enhancer elements, and co‐transfected these reporter plasmids into HEK‐293T cells along with wild‐type YAP or its mutants: YAP‐S127A (constitutively active) and YAP‐S94A (TEAD‐binding deficient) (Figure S5A, Supporting Information). Dual‐luciferase reporter assays revealed that both the promoter and E1/E2 enhancer regions were robustly activated by wild‐type YAP and YAP‐S127A, while the YAP‐S94A mutant failed to induce significant transcriptional activity (Figure 4C). Furthermore, mutation of the TEAD4 binding motifs within the promoter and enhancer regions completely abolished YAP‐induced transcription activity (Figure 4D), indicating that YAP regulates *AJUBA* transcription through TEAD‐dependent activation of its super‐enhancer.

Next, we investigated whether YAP could directly activate the *AJUBA* super‐enhancer. As previously reported, active super‐enhancers can be transcribed into non‐coding RNAs, termed seRNAs (super‐enhancer RNAs), and the abundance of seRNAs serves as a surrogate marker of SE activity.^[^ ^35^, ^36^ ^]^ To detect *AJUBA*‐associated seRNA (*seAJUBA*), we first identified its putative transcriptional region using the FANTOM5 database (Figure S5B, Supporting Information) and performed a rapid amplification of cDNA ends (RACE) assay to define its boundaries (Figure S5C, Supporting Information). Based on this information, we designed specific probes for RNA‐fluorescence in situ hybridization (RNA‐FISH) and primers for RT‐qPCR. RNA‐FISH and qPCR analyses revealed that overexpression of wild‐type YAP, but not the TEAD‐binding‐deficient mutant YAP‐S94A, significantly increased *AJUBA* seRNA levels in MCF‐7 cells. Conversely, *YAP* knockdown markedly reduced *AJUBA* seRNA expression in MDA‐MB‐231 cells (Figure 4E–H). These results indicate that YAP directly activates the *AJUBA* super‐enhancer in a TEAD‐dependent manner, as evidenced by its ability to induce *seAJUBA* transcription.

Promoters and enhancers are known to form spatial chromatin loops to facilitate transcription initiation.^[^ ^37^ ^]^ Thus, we sought to determine whether YAP enhances the physical interaction between the *AJUBA* super‐enhancers (E1/E2) and their promoter region. High‐throughput chromosome conformation capture (Hi‐C) data from the ENCODE database revealed putative chromatin–chromatin interactions between the *AJUBA* promoter and the E1/E2 enhancer regions, suggesting the presence of a transcriptionally active chromatin loop at this locus (Figure 4I). Consistently, luciferase reporter assays demonstrated that co‐construction of E1/E2 enhancer elements plus the *AJUBA* promoter led to significantly higher transcriptional activity in HEK‐293T cells overexpressing YAP, compared to the promoter or E1/E2 enhancer alone (Figure 4J). These findings indicate that YAP promotes enhancer‐promoter interaction, thereby facilitating *AJUBA* transcriptional activation.

To further investigate how super‐enhancers contribute to YAP‐induced *AJUBA* transcription and spatial interaction of *AJUBA* enhancer‐promoter loop, we employed the CRISPR affinity purification in situ of regulatory elements (CAPTURE) technique^[^ ^38^, ^39^ ^]^ to examine protein occupancy and chromatin interactions at the *AJUBA* super‐enhancer. In our study, a FLAG‐biotin (FB)‐tagged dCas9 and BirA biotin ligase were guided to the *AJUBA* super‐enhancer region using specific sgRNAs targeting the E1/E2 enhancer, respectively (sgRNA‐E1/sgRNA‐E2) (Figure 4K). Immunoprecipitation followed by western blotting demonstrated that the dCas9 complex targeted to E1/E2 could pull down YAP, TEAD4, RNA polymerase II (POLR2A), and enhancer‐associated proteins, including H3K27ac, EP300, and BRD4, in MDA‐MB‐231 cells (Figure 4L). Moreover, overexpression of wild‐type YAP, but not the TEAD‐binding‐deficient S94A mutant, enhanced the recruitment of TEAD4, EP300, BRD4, POLR2A, and acetylated H3K27 to the *AJUBA* super‐enhancer region in MCF‐7 (Figure 4M). The E1/E2 guided CAPTURE could also enrich the promoter sequence of *AJUBA*, indicating the presence of an enhancer–promoter loop (E–P loop) at the *AJUBA* SE region (Figure 4N). Consistently, wild‐type YAP overexpression significantly promoted physical interaction between the E1/E2 enhancer and the *AJUBA* promoter in MCF‐7 cells (Figure 4O). Collectively, these findings indicate that YAP activates the *AJUBA* super‐enhancer and promotes enhancer‐promoter proximity, thereby facilitating transcriptional upregulation of *AJUBA*.

### 2.5 YAP Interacts with the *AJUBA* Super‐Enhancer via Phase Separation

**Figure 5:**
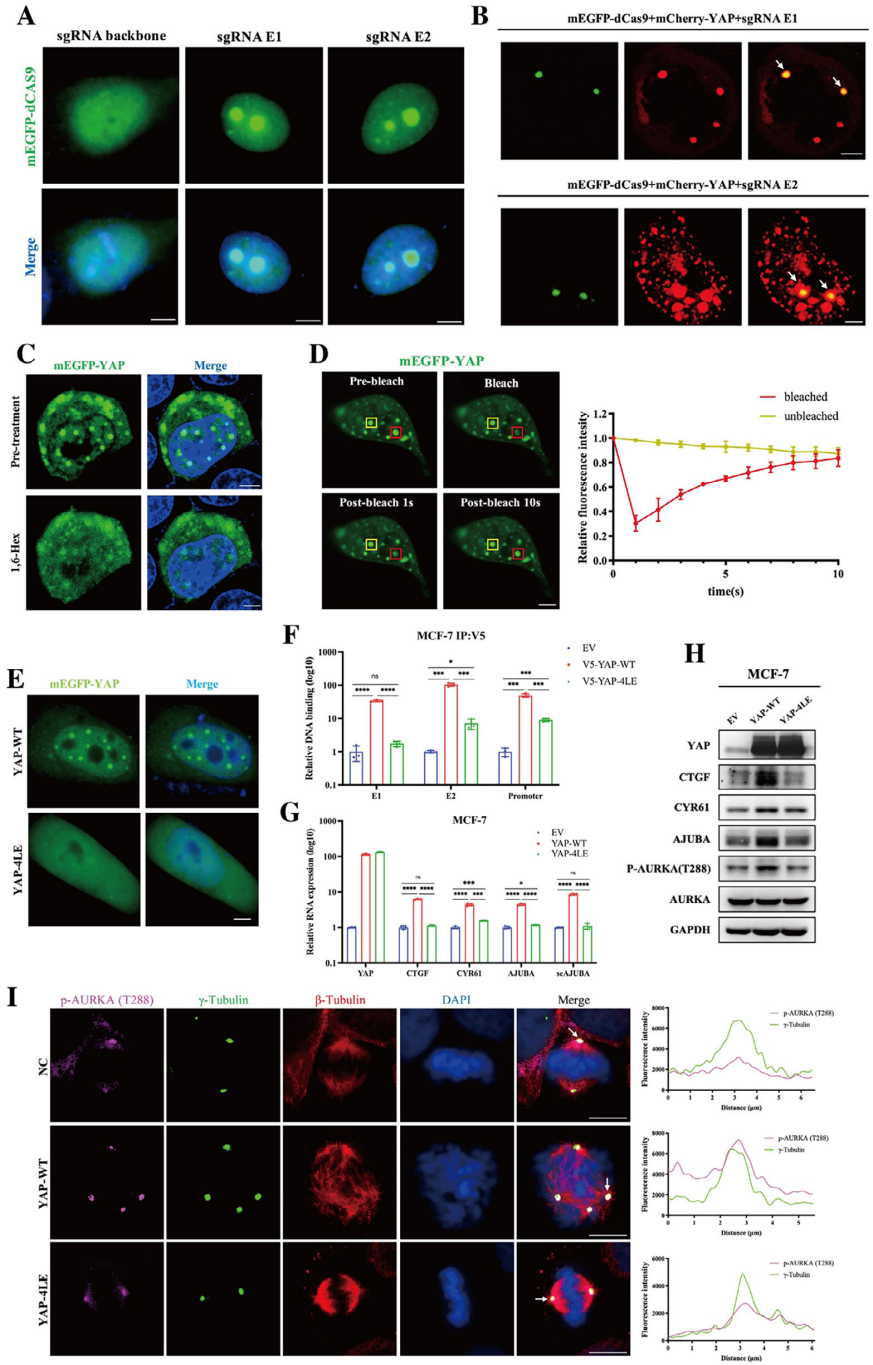
YAP interacts with the *AJUBA* super‐enhancer via phase separation. A) The efficiency of mEGFP‐dCAS9 in marking the E1/E2 loci of the *AJUBA* gene was evaluated by confocal. E1 and E2 were marked by mEGFP fused to dCAS9 (green) with targeting sgRNAs. Hoechst 33342 (blue) was used for nuclei staining. Sale bar: 5 µm. B) Colocalization of mCherry‐YAP (red) and E1 and E2 loci of the *AJUBA* gene in MCF‐7 cells was analyzed by confocal. E1 and E2 were marked by mEGFP fused dCAS9 (green) with targeting sgRNAs. Scale bar: 5 µm. C) MCF‐7 cells transfected with mEGFP‐YAP (green) were observed for the formation of YAP phase separation under confocal microscopy before and after treatment with PBS or 3% 1,6‐Hex for 30 s. Hoechst 33342 (blue) was used for nuclei staining. Scale bar: 5 µm. D) Representative images of fluorescence recovery after photobleaching (FRAP) assay in MCF‐7 cells transfected with mEGFP‐YAP (green). The bleached and control YAP condensates were marked by red and yellow rectangles, respectively (left). Quantifications of relative fluorescence intensity of bleached and control YAP condensate at the indicated time point were shown (right), data were shown as mean ± SEM. Each experiment was repeated in triplicates, scale bar: 5 µm. E) mEGFP fused wild‐type YAP (YAP WT) or mEGFP fused 4LE mutant YAP (YAP 4LE) were transiently transfected into MCF‐7 cells, and the representative images of YAP protein phase separation were shown. Scale bar: 5 µm. F) The binding capacity of wild‐type YAP (V5‐YAP‐WT) or 4LE mutant YAP (V5‐YAP‐4LE) to E1/E2 or promoter on *AJUBA* gene in MCF‐7 cells was evaluated via ChIP assay and quantified by qPCR. An empty vector was used as a negative control. Each experiment was repeated in triplicates, data were shown as mean ± SEM. ns, not statistically significant; *p < 0.05; ***p < 0.001; ****p < 0.0001. G) MCF‐7 cells transfected with EV, YAP‐WT, and YAP‐4LE plasmids were collected for qPCR, *CTCF*, *CYR61*, *AJUBA* mRNA, and seRNA (*seAJUBA*) expression levels were detected. *GAPDH* was used as an endogenous control. Each experiment was repeated in triplicates, data were shown as mean ± SEM. ns, not statistically significant; *p < 0.05; ***p < 0.001; ****p < 0.0001. H) MCF‐7 cells transfected with EV, YAP‐WT, and YAP‐4LE plasmids were collected for immunoblot. Cell lysates were probed for YAP, CTGF, CRY61, AJUBA, AURKA, and p‐AURKA (T288). GAPDH was used as a loading control. I) Representative images showed AURKA activation at the centrosome via immunofluorescence in MCF‐7 stable cells with YAP‐WT or YAP‐4LE overexpression. p‐AURKA (T288) was stained with Alexa Fluor 647 (violet), γ‐Tubulin was stained with Dylight 488 (green), β‐Tubulin was stained with Alexa Fluor 555 (red), and Nuclei were stained with DAPI (blue). Scale bar: 10 µm. Line plot analyzed the colocalization and fluorescence intensity of p‐AURKA (T288) and γ‐Tubulin.

A previous study has shown that YAP can undergo LLPS in the nucleus, thereby promoting transcriptional activation of downstream target genes.^[^ ^40^ ^]^ To explore whether YAP utilizes LLPS to activate the *AJUBA* super‐enhancer, we visualized the chromatin‐bound E1/E2 region in MCF‐7 cells using the mEGFP‐tagged dCas9 (**Figure** **5**A). Notably, when mCherry‐tagged YAP was co‐transfected, we observed the formation of YAP condensates that co‐localized with the dCas9‐targeted E1/E2 loci (Figure 5B), suggesting spatial association between YAP and the *AJUBA* enhancer.

Further characterization revealed that exogenously expressed mEGFP‐YAP formed droplet‐like nuclear condensates in MCF‐7 cells, which exhibited hallmark features of LLPS. These condensates were disrupted following treatment with 1,6‐hexanediol (Figure 5C) and demonstrated dynamic molecular exchange, as evidenced by fluorescence recovery after photobleaching (FRAP) (Figure 5D). These findings support the hypothesis that YAP undergoes phase separation at the *AJUBA* super‐enhancer.

To further investigate the function of YAP LLPS at the *AJUBA* super‐enhancer, we utilized a previously characterized mEGFP‐tagged YAP C‐terminal coiled‐coil (C‐C) domain mutant (4LE), which has been reported to be defective in YAP LLPS.^[^ ^40^ ^]^ As expected, the 4LE mutant YAP failed to form droplet‐like condensates, confirming the loss of LLPS capacity (Figure 5E). Importantly, the 4LE mutant also abolished YAP‐mediated chromatin binding of the E1/E2 enhancer and *AJUBA* promoter (Figure 5F), and significantly impaired the transcription of *AJUBA* mRNA and seRNA, as well as classical YAP/TAZ downstream genes, including *CTGF* and *CYR61*, in MCF‐7 cells (Figure 5G). In addition, the 4LE mutant failed to induce AJUBA protein expression and downstream AURKA activation (Figure 5H,I). These results indicate that YAP's phase separation capability is essential for activating the *AJUBA* super‐enhancer, thereby facilitating *AJUBA* transcription and AURKA‐hyperactivation‐dependent aberrant mitosis.

### 2.6 The *AJUBA* Super‐Enhancer is Essential for YAP‐Induced Aberrant Mitosis

To further evaluate the functional importance of the *AJUBA* super‐enhancer in YAP‐mediated transcriptional regulation, we first treated MCF‐7 and MDA‐MB‐231 cells with JQ‐1, a pan‐BET bromodomain inhibitor known to broadly suppress enhancer activity.^[^ ^41^ ^]^ JQ‐1 treatment led to a dose‐dependent decrease in AJUBA protein level, consistent with changes in H3K27ac (Figure S5D,E, Supporting Information), suggesting that enhancer activity is required for maintaining AJUBA expression.

**Figure 6:**
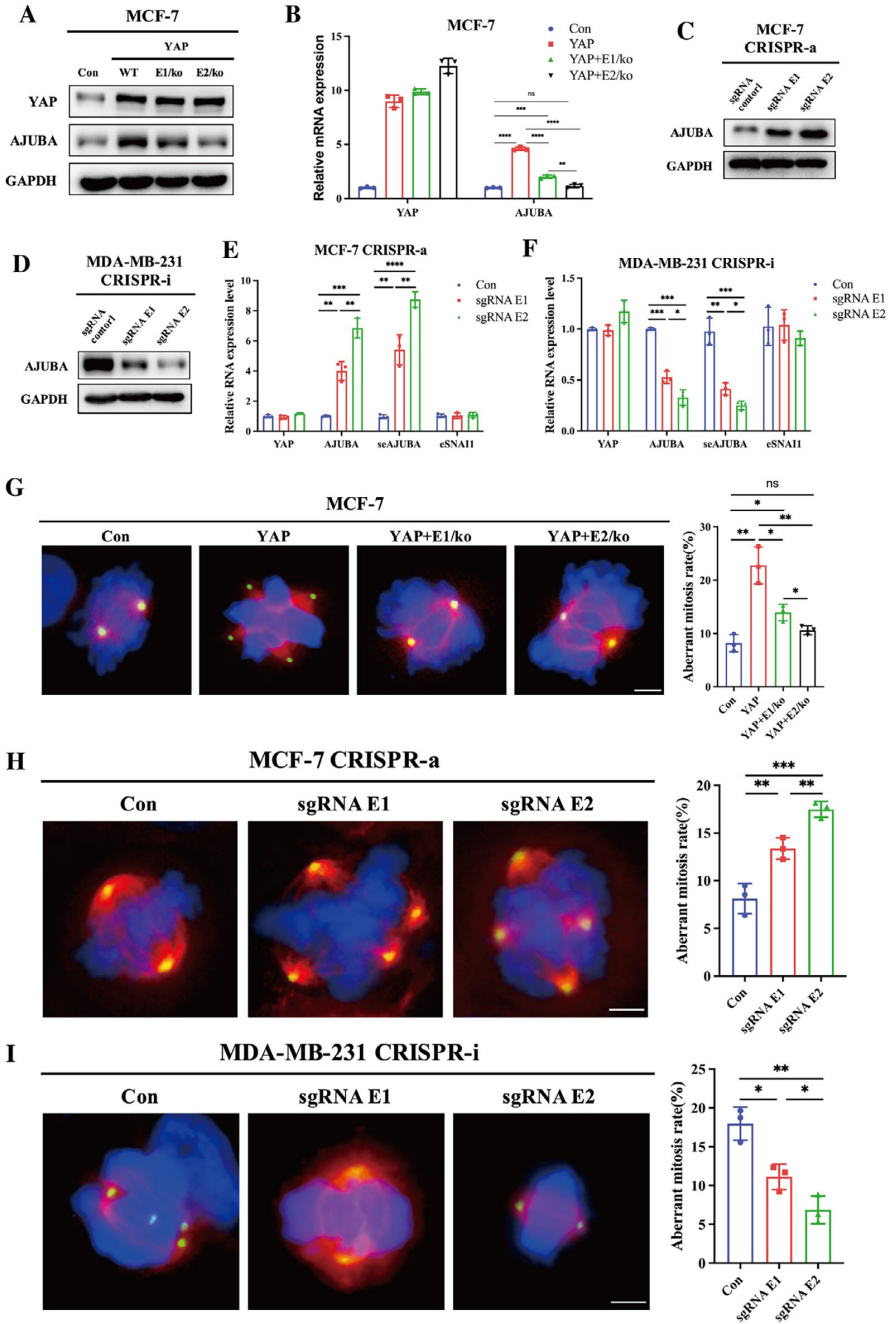
The *AJUBA* super‐enhancer is essential for YAP‐induced aberrant mitosis. A) AJUBA protein levels in MCF‐7 with control (MCF‐7 Con), YAP overexpression (MCF‐7 YAP‐WT), YAP overexpression plus E1 knockout (YAP+E1/ko), and YAP overexpression plus E2 knockout (YAP+E2/ko) stable cells were examined by western blotting. GAPDH was used as an endogenous control. B) *AJUBA* mRNA expression level in MCF‐7 with control (MCF‐7 Con), YAP overexpression (MCF‐7 YAP), YAP overexpression plus E1 knockout (YAP+E1/ko), and YAP overexpression plus E2 knockout (YAP+E2/ko) stable cells was detected. *GAPDH* was used as an endogenous control. ns, not statistically significant; **p < 0.01; ***p < 0.001; ****p < 0.0001. C) CRISPR activation assay (CRISPR‐a) was performed in MCF‐7 cells using sgRNA control, sgRNAs targeting E1 (sgRNA E1) or sgRNAs targeting E2 (sgRNA E2) on the *AJUBA* locus. AJUBA protein levels were examined by western blotting. GAPDH was used as a loading control. D) CRISPR interference assay (CRISPR‐i) was performed on MDA‐MB‐231 cells using an sgRNA control, sgRNAs targeting E1 (sgRNA E1), or sgRNAs targeting E2 (sgRNA E2) at the *AJUBA* locus. AJUBA protein levels were examined by western blotting. GAPDH was used as a loading control. E) CRISPR activation assay (CRISPR‐a) was performed in MCF‐7 cells using sgRNA control (Con), sgRNAs targeting E1 (sgRNA E1), or sgRNAs targeting E2 (sgRNA E2) sequence on the *AJUBA* locus. The *AJUBA* mRNA and seRNA (*seAJUBA*) expression levels were quantified via qPCR assay. *SANI1* seRNA (*eSNAI1*) was used as a negative control, and *GAPDH* was used as an endogenous control. **p < 0.01; ***p < 0.001, ****p < 0.0001. F) CRISPR interference assay (CRISPR‐i) was performed in MDA‐MB‐231 cells using sgRNA control (Con), sgRNAs targeting E1 (sgRNA E1), or sgRNAs targeting E2 (sgRNA E2) sequence on the *AJUBA* locus. The *AJUBA* mRNA and seRNA (*seAJUBA*) expression levels were quantified via qPCR assay. *SANI1* seRNA (*eSNAI1*) was used as a negative control, and *GAPDH* was used as an endogenous control. *p < 0.05; **p < 0.01; ***p < 0.001. G–I) Aberrant mitosis was observed by immunofluorescence in the indicated MDA‐MB‐231 and MCF‐7cells. β‐Tubulin was stained with Alexa Fluor 555 (red), γ‐Tubulin was stained with Dylight 488 (green), and Nuclei were stained with DAPI (blue). Representative images were shown and scale bar: 10 µm. For each experiment, 60–80 mitotic cells were counted, and three independent experiments were performed. Histograms showed the mean percentage ± SD of aberrant mitosis rate. ns, not statistically significant; *p < 0.05; **p < 0.01; ***p < 0.001. G) MCF‐7 with control (MCF‐7 Con), YAP overexpression (MCF‐7 YAP), YAP overexpression plus E1 knockout (YAP+E1/ko), and YAP overexpression plus E2 knockout (YAP+E2/ko) stable cells. H) CRISPR‐a performed in MCF‐7 cells using sgRNA control, sgRNAs targeting E1 (sgRNA E1), or sgRNAs targeting E2 (sgRNA E2) on the *AJUBA* locus. I) CRISPR‐I performed on MDA‐MB‐231 cells using an sgRNA control, sgRNAs targeting E1 (sgRNA E1), or sgRNAs targeting E2 (sgRNA E2) at the *AJUBA* locus.

To directly assess the role of the *AJUBA* super‐enhancer, we employed CRISPR/Cas9‐mediated deletion of the E1/E2 enhancer elements using pre‐designed sgRNAs (Figure S5F, Supporting Information). Disruption of E1/E2 significantly abolished YAP‐induced *AJUBA* transcription in MCF‐7 cells (**Figure** **6**A,B). Furthermore, CRISPR‐mediated activation (CRISPR‐a) of E1/E2 in MCF‐7 cells markedly upregulated *AJUBA* and *seAJUBA* expression, whereas interference (CRISPR‐i) with the same regions in MDA‐MB‐231 cells suppressed *AJUBA* mRNA and *seAJUBA* levels (Figure 6C–F).

To further confirm the critical role of the *AJUBA* super‐enhancer in YAP‐induced aberrant mitosis, we first examined mitotic abnormalities in MCF‐7 cells with CRISPR‐mediated deletion of the E1/E2 enhancer elements. The results showed that E1/E2 knockout effectively reversed YAP‐induced mitotic defects, including the formation of supernumerary centrosomes and defective mitosis (Figure 6G). Consistently, CRISPR activation (CRISPRa) of E1/E2 in MCF‐7 cells promoted mitotic abnormalities (Figure 6H). Conversely, CRISPR interference (CRISPRi) targeting the E1/E2 region significantly reduced the frequency of aberrant mitoses in MDA‐MB‐231 cells (Figure 6I), further supporting the functional relevance of this super‐enhancer region. Taken together, these findings demonstrate that YAP induces aberrant mitosis and centrosome amplification by activating the *AJUBA* super‐enhancer, thereby establishing a mechanistic link between enhancer activation and chromosomal instability in breast cancer cells.

### 2.7 Depletion of the *AJUBA* Super‐Enhancer Suppresses YAP‐Induced Aberrant Mitosis and Tumor Progression

Given the relatively strong regulatory activity of the E2 enhancer element within the *AJUBA* super‐enhancer, we next evaluated its biological significance in YAP‐driven tumorigenesis in vivo. Xenograft models were established by orthotopically injecting MCF‐7 cells into female NOD/SCID mice under four conditions: control, YAP overexpression, E2 knockout, and YAP overexpression combined with E2 knockout.

**Figure 7:**
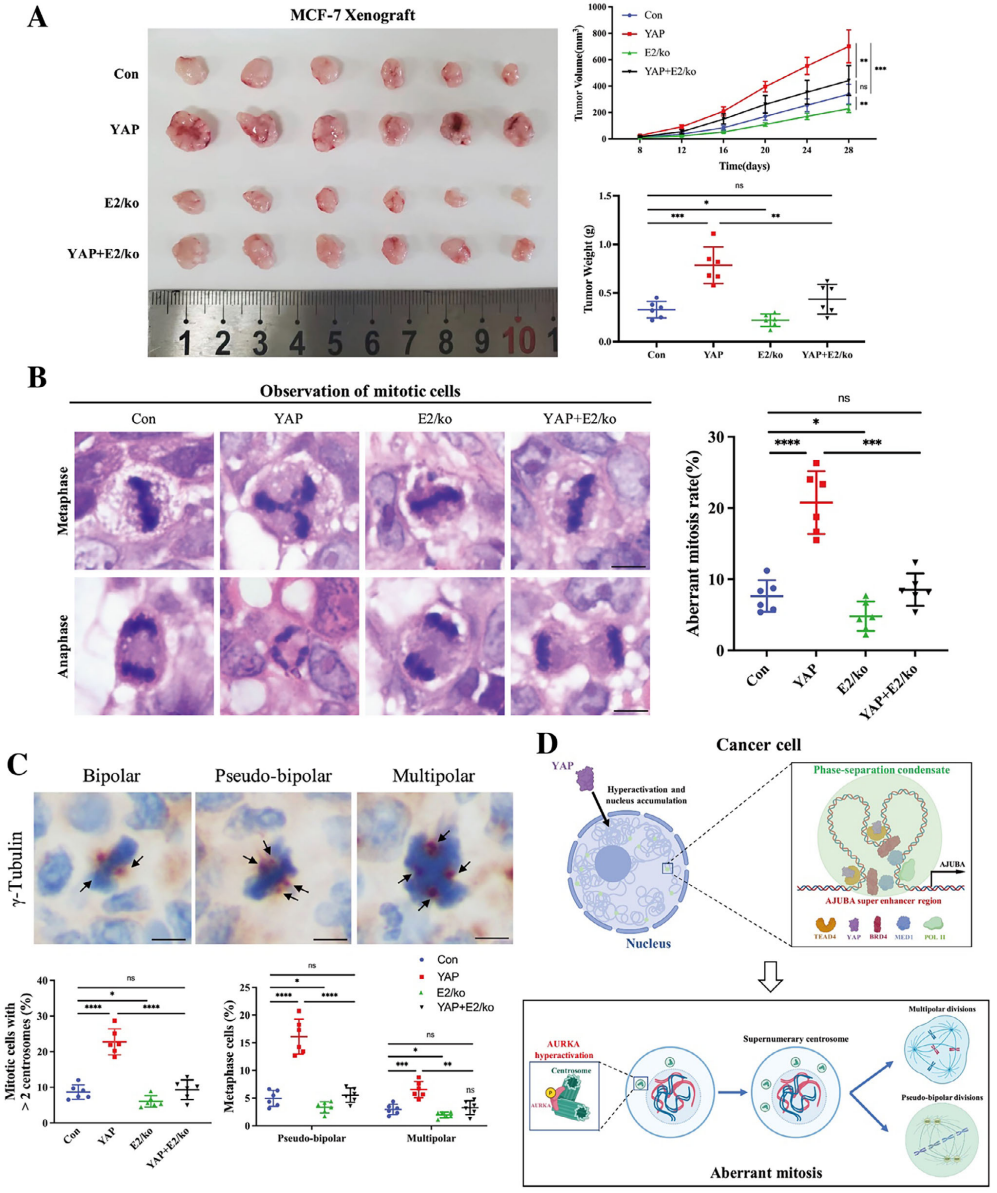
Depletion of the *AJUBA* super‐enhancer suppresses YAP‐induced aberrant mitosis and tumor progression. A) MCF‐7 with control (MCF‐7 Con), YAP overexpression (MCF‐7 YAP), E2 knockout (E2/ko), and YAP overexpression plus E2 knockout (YAP+E2/ko) stable cells were orthotopically implanted into BALB/c nude mice (*n* = 6). Tumor growth curves and weights were measured as indicated. Data were presented as mean ± SD. ns, not statistically significant; *p < 0.05; **p < 0.01; ***p < 0.001. B) H&E staining and observation of aberrant mitosis in xenograft tumors from (A). Scale bar: 4 µm. Scatterplots showed the mean percentage ± SD of aberrant mitosis rate. ns, not statistically significant; *p < 0.05; ***p < 0.001; ****p < 0.0001. C) Xenograft tumors form (A) were immunohistochemistry stained with γ‐tubulin. Representative images of pseudo‐bipolar, multipolar mitosis and centrosome numbers were shown (scale bar: 4 µm). Arrows pointed to the centrosomes. For each sample, 60–80 metaphase cells were counted. Data were presented as mean percentage ± SD in scatterplots. ns, not statistically significant; *p < 0.05; **p < 0.01; ***p < 0.001; ****p < 0.0001. D) Proposed model for YAP phase separation inducing *AJUBA* expression and resulting in aberrant mitosis in cancer cells.

Four weeks post‐implantation, tumor tissues were harvested and analyzed. Tumor volume measurements and immunofluorescence stain of Ki67 showed that E2 knockout significantly suppressed tumor growth and cell proliferation in MCF‐7 cells with YAP‐overexpression (**Figure** **7**A; Figure S5G, Supporting Information). Histological analysis further revealed a marked reduction in the frequency of aberrant mitoses in tumors exhibiting E2 deletion within the context of YAP overexpression (Figure 7B). Moreover, E2 knockout attenuated YAP‐induced mitotic defects, including pseudo‐bipolar and multipolar divisions and the presence of supernumerary centrosome clusters during mitosis (Figure 7C).

Collectively, these findings support a model in which the YAP/TEAD complex is recruited to the *AJUBA* super‐enhancer region via phase separation, thereby activating *AJUBA* transcription, stimulating AURKA signaling, and ultimately promoting mitotic aberrations and aneuploidy (Figure 7D).

## 3 Discussion

Genomic instability is a major driver of tumor heterogeneity and evolution^[^ ^42^ ^]^ and is often positively correlated with malignant progression, chemotherapy resistance, and poor prognosis.^[^ ^43^, ^44^ ^]^ Existing studies indicate that YAP‐drive chromosomal instability represents a major source of tumor progression,^[^ ^10^, ^16^, ^45^ ^]^ while the direct mechanisms on how YAP dysfunction leads to the generation of cells harboring chromosomal abnormalities remain poorly understood.

The objective of this study was to elucidate the mechanisms by which aneuploid cells arise following YAP hyperactivation. In malignancies, several factors have been implicated in mitotic abnormalities, including weakened spindle checkpoint signaling, supernumerary centrosomes, defects in chromatid cohesion, abnormal kinetochore‐microtubule attachments, and increased spindle microtubule dynamics.^[^ ^46^ ^]^ Although previous studies have reported that YAP transcriptionally activates CIN‐related genes,^[^ ^16^ ^]^ it remains unclear whether this activation represents a cause or consequence of chromosomal instability. Notably, the expression of these genes can also be induced by the aneuploid state itself.^[^ ^47^ ^]^ In the present study, we observed that YAP activation promotes supernumerary centrosome clustering in breast cancer cells, leading to abnormal chromosomal segregation and aneuploidy. Specifically, YAP hyperactivation directly induces AURKA phosphorylation at centrosomes, thereby promoting abnormal pseudo‐bipolar and multipolar division. Collectively, our findings not only reinforce the role of YAP in driving chromosomal instability but also uncover a previously unrecognized biological function of YAP in regulating centrosome‐spindle assembly and defective mitosis.

Tetraploidization has been implicated in tumorigenesis across multiple human cancers.^[^ ^48^ ^]^ The tetraploid intermediate, typically characterized by the presence of extra centrosomes, often undergoes defective mitosis, giving rise to aneuploid daughter cells.^[^ ^26^, ^49^ ^]^ In general, chromosomal instability drives intratumoral heterogeneity; however, excessive CIN can lead to catastrophic chromosome segregation errors, thereby functioning as a tumor‐suppressive mechanism.^[^ ^1^ ^]^ Near‐triploid and near‐tetraploid cancer cells appear to better tolerate aneuploidy than diploid cells. Nevertheless, the threshold of tolerable chromosomal variation remains undefined, underscoring the need to elucidate the mechanisms by which cancer cells achieve an optimal balance between tolerance and restraint during aberrant mitosis.^[^ ^6^, ^11^, ^50^ ^]^

YAP is well recognized for its role in driving cell proliferation and tumor growth; notably, our findings demonstrate that its overexpression also provokes profound mitotic defects, including centrosome amplification and multipolar spindle formation. This paradox highlights the context‐dependent role of CIN in tumor evolution. Specifically, we show that YAP not only promotes multipolar divisions but also induces pseudo‐bipolar divisions, thereby enabling continued proliferation despite segregation errors. Importantly, cancer cells undergoing multipolar mitosis can still generate viable progeny that inherit aneuploid karyotypes.^[^ ^28^ ^]^ Together with its established pro‐proliferative function,^[^ ^51^ ^]^ these results suggest that YAP‐driven aberrant mitosis accelerates the accumulation of aneuploidy, fueling CIN and promoting tumor evolution.

Our results also revealed that YAP can directly induce *AJUBA* transcription, thereby promoting hyperactivation of Aurora A kinase (AURKA), a key enzyme involved in microtubule nucleation during centrosome maturation and spindle assembly.^[^ ^52^, ^53^ ^]^ *AJUBA* encodes a LIM domain‐containing scaffold protein that interacts with AURKA, facilitating its autophosphorylation at T288^[^ ^54^ ^]^ and subsequent activation. Notably, AJUBA acts upstream of the Hippo pathway by inactivating the key Hippo pathway kinases, large tumor suppressor kinases 1/2 (*LATS1/2*). However, depletion of YAP could also downregulate AJUBA expression.^[^ ^55^, ^56^ ^]^ In our study, manipulation of AJUBA exerted only a modest effect on YAP/TAZ expression, at least in breast cancer cells. Further investigation into the regulatory interplay between YAP, AJUBA, and AURKA may identify novel vulnerabilities for therapeutic intervention in YAP‐driven cancers.

Traditionally, YAP/TAZ are thought to regulate downstream gene expression primarily by interacting with transcription factors such as TEADs, RUNX, and TEFs, thereby activating the promoters of target genes.^[^ ^57^ ^]^ However, emerging researches show that enhancers may play a more important role in Hippo‐related tumorigenesis and progression.^[^ ^57^, ^58^ ^]^ Zhu et al. reported that YAP acts as a co‐regulator in the enhancer region of estrogen‐regulated genes.^[^ ^59^ ^]^ Our previous study also revealed that YAP induces *TIAM1* expression through its enhancer and promotes invadopodia formation in breast cancer.^[^ ^25^ ^]^ Moreover, YAP/TAZ have been shown to associate with a large set of enhancers exhibiting super‐enhancer (SE)‐like functional properties.^[^ ^22^, ^24^ ^]^ SEs can spatially interact with promoters to form SE‐promoter loops that maximize transcriptional activating effects.^[^ ^60^ ^]^ In the present study, we found that the YAP/TEAD4 complex formed a transcriptional apparatus at the super‐enhancer region of *AJUBA*, strengthening the promoter‐SE loop structure and boosting *AJUBA* transcription. Notably, disruption or inhibition of the *AJUBA* super‐enhancer effectively abolished YAP‐mediated mitotic defects in breast cancer cells.

Intracellular LLPS condensates have been widely investigated.^[^ ^61^ ^]^ Considering their comparable structural nature, liquid condensate droplets can accommodate specific substrates and accelerate biochemical reactions within the compartment.^[^ ^62^ ^]^ Existing evidence has revealed that transcription factors can form LLPS condensates at SE regions, thereby strengthening SE‐promoter interactions and enhancing transcriptional activation.^[^ ^63^ ^]^ In our study, we found that YAP forms LLPS condensates at the *AJUBA* SE, which likely facilitates enhancer activation and transcriptional upregulation. Consistent with the report by Sun et al., showing that the coiled‐coil (C‐C) domain of YAP is essential for LLPS formation,^[^ ^40^ ^]^ we demonstrated that the phase separation‐deficient mutant YAP‐4LE failed to form LLPS condensates. Importantly, this mutant also abolished YAP‐induced SE/promoter interaction, resulting in impaired *AJUBA* mRNA and seRNA transcription, as well as reduced AJUBA protein expression, AURKA activation, and YAP‐mediated mitotic aberrations. These findings indicate that the phase separation capability of YAP is crucial for activating the *AJUBA* SE, thereby driving *AJUBA* transcription and AURKA‐dependent mitotic signaling.

Several important questions remain to be addressed in future studies. First, although we demonstrated that YAP induces aberrant mitosis and aneuploidy, it is intriguing that YAP overexpression promotes cell proliferation despite the high level of mitotic aberrations, which are typically associated with cell cycle arrest or cell death. In our assays, we observed that YAP not only increased multipolar divisions but also promoted pseudo‐bipolar divisions through centrosome clustering, allowing cells to proliferate despite segregation errors. Previous studies have indicated that extra centrosomes in tetraploid cells can inactivate YAP/TAZ and stabilize p53, aiding in tumor suppression.^[^ ^11^ ^]^ While in cells p53‐null or with an inactive Hippo pathway (YAP/TAZ activation), tetraploids facilitate tumor progression, which might represent one critical dynamic adaptation that allows cancer cells to tolerate supernumerary centrosomes and aberrant mitosis.^[^ ^50^, ^64^ ^]^ However, the long‐term fate of these YAP‐induced aneuploid cells and their contribution to tumor evolution remain unclear and warrant further investigation. Second, while our karyotype analysis revealed that YAP promotes chromosome number variation in cancer cells, this variation appeared to stabilize at a relatively high modal number over time, suggesting that cells may reach a new equilibrium state of aneuploidy. The potential feedback mechanisms underlying YAP‐regulated aberrant mitosis need to be further elucidated. Third, seRNAs could regulate the adjacent or distant gene transcription in both cis and trans configurations; meanwhile, those located in the cytoplasm can also mediate various cellular activities.^[^ ^36^ ^]^ Although our data demonstrated that YAP activation increases *AJUBA* seRNA transcription, the functional roles of this non‐coding RNA in Hippo signaling remain largely unknown. Future studies are required to explore the biological significance of seRNAs in YAP/TAZ‐mediated mitotic regulation and tumor progression.

In summary, our study uncovers a previously unrecognized mechanism whereby YAP induces *AJUBA* expression through phase separation at its super‐enhancer (SE) region, which in turn hyperactivates AURKA at centrosomes during M phase, leading to aberrant mitosis. These findings provide new mechanistic insight into how dysregulated YAP‐driven biomolecular condensates contribute to mitotic errors, and underscore the need for future studies on how modulating enhancer activity may suppress chromosomal instability and tumor progression in YAP‐driven cancers.

## 4 Experimental Section

### Antibodies, Reagents, Cell Lines, Datasets, and Materials

Antibodies, reagents, cell lines, datasets, and materials used in this research are listed in Table S1 (Supporting Information).

### Patient Samples

Human breast cancer tissue array, comprising primary tumor and paired para‐cancerous tissue specimens from 75 breast cancer cases, was obtained from Wuhan Baiqiandu Biotech Co. (Cat. #BRC1601). A multiple tumor tissue array containing primary tumor specimens from 37 breast, 39 lung, 38 colorectal, 38 prostate, and 38 pancreatic cancer cases was purchased from Xian Alenabio Biotech Co. (Cat. #BC000119a). Immunohistochemical staining was performed using antibodies against YAP (Cell Signaling Technology, Cat. #14 074) and AJUBA (Abcam, Cat. #ab244285) according to the manufacturers’ recommended protocols. Protein expression levels were assessed using the immunohistochemistry (IHC) score, and nuclear localization was quantified using a nuclear localization score, as previously described.^[^ ^65^ ^]^ According to the manufacturers, the collection and use of all tissue samples were approved by local institutional ethics committees, and all procedures complied with relevant ethical regulations. Ethical compliance documentation is available from the respective suppliers upon request.

### Animal Models

In this study, six‐week‐old female BALB/c nude mice were obtained from Gempharmatech Co., Ltd. (Jiangsu, China) and housed in SPF animal facilities. Animal studies were performed according to the protocols and guidelines approved by the Institutional Animal Care and Use Committee of Huazhong University of Science and Technology, Wuhan, China (approval number: TJH‐202303052). Briefly, mice were randomly assigned to different groups (*n* = 6 or 7 per group). Then, 1 × 10^6^ MDA‐MB‐231 cells or MCF‐7 cells that were manipulated as indicated were collected and resuspended in 50 µL PBS with 50% Matrigel, and then orthotopically injected into the fourth mammary fat pads of mice. Tumor size was measured every four days. Four weeks after tumor implantation, the mice were sacrificed, and the tumors were collected for further experiments.

### Cell Lines and Cell Culture

MCF‐7 and HEK‐293T cells were cultured in Dulbecco's modified Eagle's medium (DMEM), MDA‐MB‐231 cells were cultured in Leibovitz's L‐15 medium (L‐15), T‐47D and BT‐549 cells were cultured in Roswell Park Memorial Institute 1640 medium (RPMI‐1640) with 10 µg mL^−1^ human recombinant insulin. All the mediums above were supplemented with 10% fetal bovine serum and 1% penicillin/streptomycin. MCF‐10A cells were cultured using the MEGM kit.

All cells were cultured in a 5% CO_2_ incubator at 37 °C, with the exception of MDA‐MB‐231 cells which were cultured in 100% air. All cell lines were purchased from the ATCC. Verteporfin, TED‐347, MLN8237, and JQ‐1 were dissolved in DMSO and prepared as mother liquors at concentrations of 1 mm (verteporfin), 10 mm (TED‐347), 10 mM (MLN8237), and 2 mM (JQ‐1). The cells were treated with the specified drug at the appropriate working concentration and duration, and DMSO was used as a control at an equivalent volume.

### Plasmid Construction, Stable Cell Lines Establishment, and Transfection

Plasmids were constructed by ligating the DNA fragments to the indicated linearized vectors. Transient gene overexpression plasmids were conducted in the pcDNA3.1 vector, and stable overexpression plasmids were conducted in the pLVX‐Neo vector. For the Dual‐luciferase reporter assay, cis‐regulatory element sequences from the E1/E2/promoter or the indicated combination of the E1/E2/promoter were separately cloned into PGL3‐Basic (promoter, E1 + P, E2 + P, E1 + E2 + P) or pGL3‐Enhancer (E1, E2, E1 + E2) vector. For CRISPR/Cas9 knockout, indicated sgRNAs were cloned into pSpCas9(BB)‐2A‐Puro‐sgRNA vector, and for dCas9 guide CRISPR a/i, sgRNA (MS2) cloning backbone was used to carry sgRNAs. For the dCas9 guide CAPTURE system, multiple gRNAs were constructed into the sgRNA (MS2) cloning backbone and expressed in tandem using an artificial polycistronic‐tRNA‐gRNA (*PTG*) gene as previously described.^[^ ^66^ ^]^ The targeted sequences are listed in Table S2 (Supporting Information). Site‐directed mutations were constructed using the MultiS Fast Mutagenesis Kit according to the manufacturer's instructions, and primers used are listed in Table S3 (Supporting Information).

Plasmids were transfected using TurboFect or Lipofectamine 3000, while small interfering RNAs (siRNAs) were transfected using Lipofectamine 2000. The siRNAs were obtained from RiboBio Co., Ltd. (Guangzhou, China). The siRNA sequences are listed in Table S4 (Supporting Information), and plasmid information is listed in Table S1 (Supporting Information).

Stable FB‐dCas9 and BirA‐expressing or YAP‐overexpressing MCF‐7 or MDA‐MB‐231 cells were generated using a lentiviral system (pLVX, psPAX2, and pMD2.G), respectively. Lentiviruses were packaged in HEK‐293T cells, then filtered using a 0.45‐µm filter and concentrated using the Universal Virus Concentration Kit. MCF‐7 cells were infected and selected using 700 µg mL^−1^ of G418, while MDA‐MB‐231 using 1200 µg mL^−1^ of G418, for two weeks. The CRISPR/Cas9 system was used to generate the E1/ko, E2/ko, YAP+E1/ko, YAP+E2/ko MCF‐7 cell lines, and YAP KO MDA‐MB‐231 cell lines. Briefly, sgRNAs were designed using the website software E‐CRISP (http://www.e‐crisp.org/E‐CRISP/) and cloned into the pSpCas9(BB)‐2A‐Puro (PX459) V2.0 plasmid. The engineered plasmids were transfected into the indicated cells. Then, 48 h after transfection, the cells were selected under 2 µg mL^−1^ puromycin (for MCF‐7 cells) or 4 µg mL^−1^ (for MDA‐MB‐231 cells), until the cells in the control group died. Surviving cells were seeded in 96‐well plates to obtain single‐cell clones, which were collected for Sanger sequencing. All stable cell lines were verified using western blotting and qPCR.

### Phosphorylation Antibody Screening Microarray Analysis

The RayBio label‐based (L‐Series) human phosphorylation screening array kit AAH‐BLG‐PHO1 (Ray Biotech, Inc., USA) was employed to assess phosphorylation levels across 500 distinct human proteins in cell lysates. The experiment adhered to the manufacturer's protocol. Briefly, lysates from MDA‐MB‐231 WT and YAP KO cells were collected, dialyzed with dialysis buffer, labeled with biotin, and incubated overnight with arrays. Subsequently, the glass slides were treated with Cy3‐conjugated streptavidin for two hours. Detection was performed using an InnoScan 300 Microarray Scanner (Parc d'activité Activestre, Carbonne, France), and the images were analyzed using the RayBio analysis tool. Data quantification and normalization were conducted according to the manufacturer's instructions, with relative fluorescence intensity serving as the metric for evaluating changes in protein phosphorylation levels between the two groups.

### Quantitative Real‐Time Polymerase Chain Reaction (qPCR)

qPCR was performed as previously described.^[^ ^65^ ^]^ Briefly, Total RNA was extracted using TRIzol reagent, and cDNA was synthesized using the RT reagent Kit with gDNA Eraser, according to the manufacturer's instructions. qPCR was performed using TB Green Premix Ex Taq II FAST qPCR Kit on a QuantStudio 3 Real‐Time PCR Instrument (Applied Biosystems). The primers used are listed in Table S5 (Supporting Information). Each experiment was performed in triplicates.

### Immunoblot Assays

Immunoblotting assays were performed as previously described.^[^ ^65^ ^]^ Briefly, Total protein was extracted using NP40 lysis buffer supplemented with protease inhibitor cocktail, phosphatase inhibitor cocktail I, and phosphatase inhibitor cocktail II. After centrifugation, the supernatant was collected, and the protein concentration was measured using the BCA Protein Assay Kit. Samples were diluted using a 5× protein loading buffer [250 mM tris‐HCl (pH 6.8), 10% SDS, 30% glycerol, 5% β‐mercaptoethanol, and bromophenol blue], and then boiled at 95–100 °C for 5 to 10 min. Samples were loaded onto 10% SDS‐PAGE gels, separated by electrophoresis, and transferred onto PVDF membranes. Membranes were blocked in 5% non‐fat milk for 2 h at room temperature, then incubated overnight at 4 °C with the primary antibodies (see Table S1, Supporting Information) at a recommended dilution ratio according to the manufacturer's protocol. After washing, the membranes were incubated with 1:5000 horseradish peroxidase‐conjugated goat anti‐rabbit IgG at room temperature for 2 h. Finally, the membranes were visualized using a West Pico PLUS Chemiluminescent Substrate Kit.

### Chromatin Immunoprecipitation (ChIP)

ChIP was performed using a Simple ChIP Enzymatic Chromatin IP Kit according to the manufacturer's instructions. The PCR was performed using Phanta Max super‐fidelity DNA Polymerase according to the manufacturer's protocol, and the products were analyzed using electrophoresis on a 1% tris‐acetate‐EDTA (TAE)/ethidium bromide agarose gel. For ChIP‐qPCR experiments, ChIP‐enriched DNA fragments were quantified using the TB Green Premix Ex Taq II FAST qPCR Kit, as previously described. The primers used are listed in Table S6 (Supporting Information). Each experiment was performed in triplicates.

### Rapid Amplification of cDNA Ends (RACE Assay)

RACE was performed using HiScript‐TS 5′/3′ RACE Kit. Briefly, total RNA was isolated using TRIzol reagent, and first‐strand cDNA was synthesized according to the manufacturer's guidelines. Subsequently, the products were used for 5′‐RACE and 3′‐RACE, respectively, and the amplified fragments were analyzed by electrophoresis in a 1% TAE/ethidium bromide agarose gel. The corresponding gel fragments were purified using a FastPure gel DNA extraction mini Kit and sequenced. The gene specific 3′ RACE and 5′ RACE primers are listed in Table S6 (Supporting Information).

### Immunofluorescence

For cell immunofluorescence, indicated cells (5 × 10^4^) were seeded on prepared climbing slices in a 24‐well plate and cultured in a 37 °C incubator for 16 h. Cells were washed and fixed in 4% paraformaldehyde for 10 min at 4 °C followed by iced methanol for 10 min, and then blocked with blocking buffer. Coverslips were incubated with Rabbit anti‐γ‐tubulin (1:100) overnight at 4 °C, followed by incubating with Dylight 488 Goat Anti‐Rabbit IgG (1:200) at room temperature for 2 h. After washing, coverslips were then incubated with Alexa Fluor 555 Rabbit labeled anti‐β‐tubulin (1:200) and/or Alexa Fluor 647 labeled Rabbit anti‐p‐AURKA (T288) (1:100) at room temperature for 2 h. DAPI was used for the staining of nuclei. The prepared samples were observed under an inverted fluorescence microscope (Ts2R‐FL; Nikon, Tokyo, Japan) at 40× magnification, and colocalization of p‐AURKA and γ‐tubulin was observed using a confocal microscope (FV3000, Olympus) at 60× magnification. The aberrant mitosis rate was determined by calculating the percentage of pseudo‐bipolar and multipolar mitotic figures among the total number of mitotic cells (60–80 mitotic cells were counted per sample). Each experiment was performed in triplicates.

For tissue immunofluorescence of Ki67, paraffin‐embedded xenograft tumor tissue sample slices were routinely dewaxed, rehydrated, and heated in sodium citrate buffer (0.01 M, pH 6.0) for antigen retrieval. After being blocked with 5% bovine serum albumin for 2 h at room temperature, slices were then incubated with 1:200 diluted Ki67 antibody at 4 °C overnight, and followed by incubating with Cy3 Goat Anti‐Rabbit IgG (1:200) at room temperature for 2 h. DAPI was used for the staining of nuclei. The slices were observed under an inverted fluorescence microscope (Ts2R‐FL; Nikon, Tokyo, Japan).

### Chromosome Metaphase Spreading Assay

Indicated cells were treated with colchicine (0.1 µg mL^−1^, 37 °C, 3 h), digested and collected, then resuspended and incubated in KCl (0.075 M, 37 °C, 20 min). Subsequently, cells were fixed in freshly prepared methanol‐acetic acid (3:1 vol/vol) and incubated for 30 min at 37 °C. After centrifugation, the cells were resuspended in a small volume of fixative solution, dropped onto cold slides, and air‐dried. Slides were stained using the Giemsa stain Kit, and the chromosome number was analyzed using microscopy (BX53, OLYMPUS) with a 100× oil‐immersion objective. Chromosome numbers per cell were counted to calculate a modal chromosomal number (*n* = 100–120 metaphase cells per sample). The aneuploidy variation was assessed by quantifying the relative change in the modal chromosome number. Each experiment was performed in triplicates.

### H&E Staining Assay

For H&E staining, paraffin‐embedded xenograft tumor tissue sample slices were routinely dewaxed, rehydrated, and stained using an H&E staining kit, according to the manufacturer's instructions. The results were analyzed using a microscope (BX53, OLYMPUS) equipped with a 100× oil‐immersion objective. The percentage of mitotic cells with aberrant mitosis was quantified (60‐80 mitotic cells per sample).

### Immunohistochemistry

IHC staining for YAP, AJUBA, and γ‐tubulin in cancer tissue was performed according to the manufacturer's instructions. Briefly, slides were dewaxed, rehydrated, and heated in sodium citrate buffer (0.01 M, pH 6.0) for antigen retrieval. Subsequently, endogenous peroxidase was inhibited with 3% hydrogen peroxide and 0.1% sodium for 30 min, and nonspecific staining was blocked with 5% bovine serum albumin for 2 h at room temperature. The slides were subsequently incubated with 1:200 diluted YAP, 1:100 diluted AJUBA, or 1:800 diluted γ‐tubulin antibodies at 4 °C overnight, respectively, followed by incubating with biotinylated secondary antibodies at room temperature for 2 h. The slides were then stained using a DAB horseradish peroxidase immunohistochemistry Kit and counterstained with hematoxylin. The slides were analyzed using a microscope (BX53, OLYMPUS).

Immunohistochemically stained tissue arrays were scored separately by two experienced pathologists. The expression of YAP and AJUBA was evaluated using an IHC score, which was calculated by multiplying the proportion and intensity scores. The proportion score represents the proportion of positively stained cells: 0 (<5%), 1 (5–25%), 2 (26–50%), 3 (51–75%), and 4 (>75%). The intensity score reflected the staining intensity (0, no staining; 1, weak; 2, moderate; 3, strong). An IHC score of ≤5 was assessed as low expression, and scores of 6‐12 were evaluated as high.

### Dual‐Luciferase Reporter Assay

A dual‐luciferase reporter assay was performed using the dual‐luciferase reporter assay kit. Briefly, pGL3‐Basic/Enhancer plasmids with the inserted targeting sequences, the pRL‐TK plasmid, and the indicated YAP plasmids were co‐transfected into HEK‐293T cells. After 48 h, the cells were lysed to collect the supernatant, and firefly luciferase activity was assayed and normalized to the Renilla luciferase. Each experiment was performed in triplicates.

### In Situ CAPTURE

CRISPR affinity purification in situ of regulatory elements (in situ CAPTURE) was performed as previously described.^[^ ^38^, ^67^ ^]^ Briefly, FB‐dCas9 and BirA‐expressing stable cells transfected with *AJUBA* super‐enhancer E1/E2 sgRNAs or non‐targeting sgRNA constructed plasmids were cross‐linked with 1% formaldehyde for 10 min and quenched with 0.25 M glycine for 5 min. Cells were lysed and centrifuged to isolate the nuclei, then resuspended, and sonicated into segments of 200–500 bp in length. Supernatant was then incubated with Streptavidin T1 Dynabeads at 4 °C overnight and followed by washing with low‐salt buffer and high‐salt buffer.

To obtain dCas9‐captured DNA, chromatin fragments were eluted, reverse cross‐linked, and purified using a Simple ChIP Enzymatic Chromatin IP Kit. The products were amplified and analyzed using electrophoresis on a 1% TAE/ethidium bromide agarose gel or subjected to qPCR to detect the captured fragments.

For the obtention of dCas9‐captured proteins, the Streptavidin T1 Dynabeads were washed with IP binding buffer and suspended in 1× protein loading buffer, then incubated at 95–100 °C for 20 min. The proteins were separated by SDS‐PAGE and analyzed by Western blot. The sgRNA sequences and primers used are listed in Table S2 (Supporting Information). Each experiment was performed in triplicates.

### CRISPR Activation/Interference (CRISPR a/i)

CRISPR activation/interference (CRISPR a/i) was performed as described previously.^[^ ^25^ ^]^ Briefly, sgRNAs targeting the *AJUBA* super‐enhancer E1/E2 were cloned into an sgRNA (MS2) cloning backbone plasmid. For CRISPR activation or interference, SP‐dCas9‐VPR or dCas9‐KRAB‐MeCP2 was co‐transfected with the constructed sgRNA plasmid into MCF‐7 or MDA‐MB‐231 cells, respectively. After 48 h, cells were harvested for western blotting or qPCR analysis. The sgRNA sequences are listed in Table S2 (Supporting Information). Each experiment was performed in triplicates.

### Live Cell Imaging and Fluorescence Recovery after Photobleaching (FRAP)

For live cell imaging, 4 × 10^4^ MCF‐7 cells were seeded in a 24‐well glass‐bottom confocal plate and transfected with the mEGFP‐YAP plasmid. For 1,6‐hexanediol treatment, the indicated cells were treated with PBS or 3% 1,6‐hexanediol, and real‐time status changes in mEGFP‐YAP phase‐separation condensates were observed. Images were acquired using a confocal microscope (FV3000, Olympus) equipped with a 100× oil immersion objective and a cell culture system.

For FRAP based on live cell imaging, a YAP phase‐separation condensate was identified, and a region of interest (ROI) was drawn using Olympus FV3000 imaging software within the stimulation module. Then, the ROI was subjected to photobleaching using the 488 nm laser line at 20% laser power for 250 ms, and images were collected every 1 s post‐bleaching. Fluorescence intensity was measured using ImageJ software. FRAP experiments were performed in triplicates.

For colocalization analysis, cells were prepared as previously described and co‐transfected with the mCherry‐YAP plasmid, mEGFP‐dCas9 plasmid, and sgRNA plasmid targeting the *AJUBA* super‐enhancer E1/E2. The cells were imaged using a confocal microscope (FV3000, Olympus) with a 100× oil immersion objective.

### RNA‐Fluorescence In Situ Hybridization (RNA‐FISH)

RNA‐FISH was performed using the RNA fluorescent in situ hybridization kit according to the manufacturer's protocol. Briefly, the cells were grown on climbing slices in a 24‐well plate and fixed with 4% paraformaldehyde at room temperature for 10 min. After permeabilized, cells were incubated with pre‐hybridization buffer for 30 min at 37 °C, and then incubated with pre‐heated hybridization buffer with FISH probe mix added at 37 °C overnight, protected from light. Subsequently, the climbing slices were washed, stained with DAPI, and sealed with an antifade mounting medium. Images were obtained using an inverted fluorescence microscope (Ts2R‐FL; Nikon, Tokyo, Japan) with a 60× objective. FISH probes targeting the *AJUBA* seRNA were designed and synthesized by Guangzhou RiboBio Co., Ltd. Each experiment was performed in triplicates.

### ChIP‐Seq and High‐Throughput Chromosome Conformation Capture (Hi‐C) Data Analysis

ChIP‐seq data for H3K4me1, H3K4me3, H3K27ac, CTCF, POLR2A, and TEAD4 in MCF‐7 cells were obtained from the ENCODE database (https://www.encodeproject. org/). The ROSE algorithm was used to identify SEs from the H3K27ac ChIP‐seq data.^[^ ^68^ ^]^ Sequencing data were aligned to the human reference genome (GRCh38) using Bowtie2 and Bedtools, and the bigwig‐format files of the ChIP‐seq data were visualized using Integrative Genomics Viewer. Raw Hi‐C data for MCF‐7 cells were obtained from the ENCODE database. Quality control procedures were applied to the raw data, including the removal of low‐quality reads and appropriate trimming to ensure data quality. Clean reads were then processed using the HiC‐Pro pipeline according to the instructions.^[^ ^69^ ^]^ Subsequently, the reads were mapped to the human reference genome (GRCh38), and the identified chromatin loops were visualized using the WashU EpiGenome Browser (http://epigenomegateway.wustl.edu). The data obtained from the ENCODE database are provided in Table S1 (Supporting Information).

### High‐Throughput RNA‐Seq Analysis

All sequencing and bioinformatics analyses were conducted by OE Biotech Co., Ltd. (Shanghai, China). Briefly, total RNA was extracted from MDA‐MB‐231 cells transfected with either control siRNA (siNC) or *YAP*‐targeting siRNA (siYAP), with three biological replicates per group. RNA extraction was performed using the mirVana miRNA Isolation Kit (Ambion, USA) following the manufacturer's instructions. Strand‐specific mRNA libraries were constructed using the TruSeq Stranded mRNA LT Sample Prep Kit (Illumina, USA) according to the manufacturer's protocol. The libraries were sequenced on an Illumina NovaSeq 6000 platform to generate 150 bp paired‐end reads. Raw sequencing reads were first processed with Trimmomatic to remove adapter sequences and low‐quality bases. Clean reads were then mapped to the human reference genome (GRCh38/hg38) using HISAT2. Gene‐level quantification was performed using HTSeq‐count, and FPKM values were calculated using Cufflinks. Differentially expressed genes (DEGs) between the siYAP and siNC groups were identified using the DESeq2 R package. Functional enrichment analyses were conducted using R based on the hypergeometric distribution.

### Bioinformatic Analysis

ChIP‐seq data for YAP and TEAD4 in MDA‐MB‐231 cells were obtained from the GEO database (GSE66081) and annotated using the ChIPseeker tool. The expression profiles of siYAP/TAZ versus siNC in MDA‐MB‐231 cells were downloaded from the GEO database (GSE66082). The different expression genes with fold change ≥ 2 and *p* < 0.01 were intersected with the genes from ChIP‐seq data containing both YAP binding peaks and TEAD4 binding peaks. Gene Ontology (biological process) enrichment of these intersecting genes was performed using the DAVID software.

To identify super‐enhancer regions associated with the *AJUBA* gene, H3K27ac ChIP‐seq data from MCF‐7 cells obtained from the ENCODE database were analyzed. Peak calling was performed using MACS2, and the resulting enhancer regions were analyzed using the ROSE algorithm (https://bitbucket.org/young_computation/rose). Adjacent enhancer peaks within 12.5 kb were stitched together, and regions within ±2.5 kb of transcription start sites were excluded to avoid promoter‐associated signal. Enhancers were ranked based on H3K27ac signal intensity, and those above the inflection point were designated as super‐enhancers.

To identify potential super‐enhancer RNAs (seRNAs) transcribed from the *AJUBA* super‐enhancer region, the FANTOM5 CAGE (cap analysis of gene expression) dataset, which provides genome‐wide annotations of non‐coding RNAs derived from active regulatory elements, was analyzed. Genomic coordinates of the *AJUBA* super‐enhancer (as defined by ROSE) were used as the query region. Transcriptional activity within this region was examined using the FANTOM5 CAGE peak viewer (https://fantom.gsc.riken.jp/5/), and candidate non‐coding RNA transcriptional units overlapping with the E1 and E2 enhancer regions were extracted. These candidate seRNA loci were used to design primers for the RACE assay.

To assess the correlation between YAP and AJUBA expression in human cancers, the R2 genomics analysis and visualization platform (http://r2.amc.nl) were utilized. Gene expression data from the Cancer Genome Atlas (TCGA) datasets were selected for the following cancer types: breast invasive ductal carcinoma, lung squamous cell carcinoma, colon adenocarcinoma, prostate adenocarcinoma, and pancreatic adenocarcinoma. Pearson correlation analysis was performed using default parameters on the platform. Scatter plots were generated to visualize the linear relationship between *YAP* and *AJUBA* mRNA expression levels in each cancer type. Statistical significance and correlation coefficients (*R*‐values) were calculated and are reported.

### Statistical Analysis

SPSS (version 22.0) and GraphPad Prism (version 9.5.0) were used for statistical analyses. Continuous data were presented as mean ± standard deviation (SD) and statistically analyzed using Student's *t‐*test (two‐tailed) or analysis of variance (ANOVA). Enumeration data were analyzed using Fisher's exact test. Survival was analyzed using the Kaplan–Meier curve with a log‐rank test. Statistical significance was set at ns, not statistically significant; *p < 0.05; **p < 0.01; ***p < 0.001; and ****p <  0.0001.

## Conflict of Interest

The authors declare no conflict of interest.

## Author Contributions

R.Z. and Q.H. contributed equally to this work. Conceptualization was done by J.S. and D.X. Methodology was done by R.Z., Q.H., and J.S. Investigation was done by R.Z., Q.H., Z.C., W.M., H.D., Z.Q., and J.S. Visualization was done by R.Z. and J.S. Supervision was done by D.X. Original draft was written by R.Z., Q.H., and J.S. Writing the review and editing was done by R.Z., Q.H., L.L., J.S., and D.X.

## Supporting information



Supporting Information

## Data Availability

The data that support the findings of this study are available from the corresponding author upon reasonable request.
