# Supplementary material for: Phase‐Separation of YAP Mediates AJUBA Super Enhancer Activation to Promote Aberrant Mitosis in Breast Cancer
Source: Adv Sci (Weinh). 2025 Dec 8;13(8):e09341. doi: 10.1002/advs.202409341 (PMC12884790; doi:10.1002/advs.202409341)
Supplement: Supplementary file 1 — Supporting Information [file ADVS-13-e09341-s001.docx]

*SUPPLEMENTARY INFORMATIONS*

**Phase-separation of YAP Mediates AJUBA Super Enhancer Activation to Promote Aberrant Mitosis in Breast Cancer**

**Authors**

Rui Zhang ^1,2*^, Qingwen Huang ^1,2*^, Zhuo Chen^1,2^, Weijian Meng^1,2^, Hongliang Dong^1,2^, Zhihong Qi^1,2^, Liang Liu^1,2^, Jie Shen^1,2#^, Daxing Xie^1,2#^

**Affiliations**

^1^ Department of GI Surgery, Tongji Hospital, Tongji Medical College, Huazhong University of Science and Technology, Wuhan, 430030, China;

^2^ Molecular Medicine Center, Tongji Hospital, Tongji Medical College, Huazhong University of Science and Technology, Wuhan, 430030, China;

*These authors contribute equally to this work.

^#^Correspondence:

Jie Shen and Daxing Xie

Tongji Hospital, Tongji Medical College, Huazhong University of Science and Technology, 1095 Jiefang Ave. Wuhan, Hubei 430030, China.

E-mail: jieshen@tjh.tjmu.edu.cn and dxxie@ tjh.tjmu.edu.cn

**This file includes:**

Fig. S1 to S5

Tables S1 to S6

**Supplementary figures**

**Fig S1. YAP induces** **aberrant mitosis and aneuploidy in breast cancer.**

**
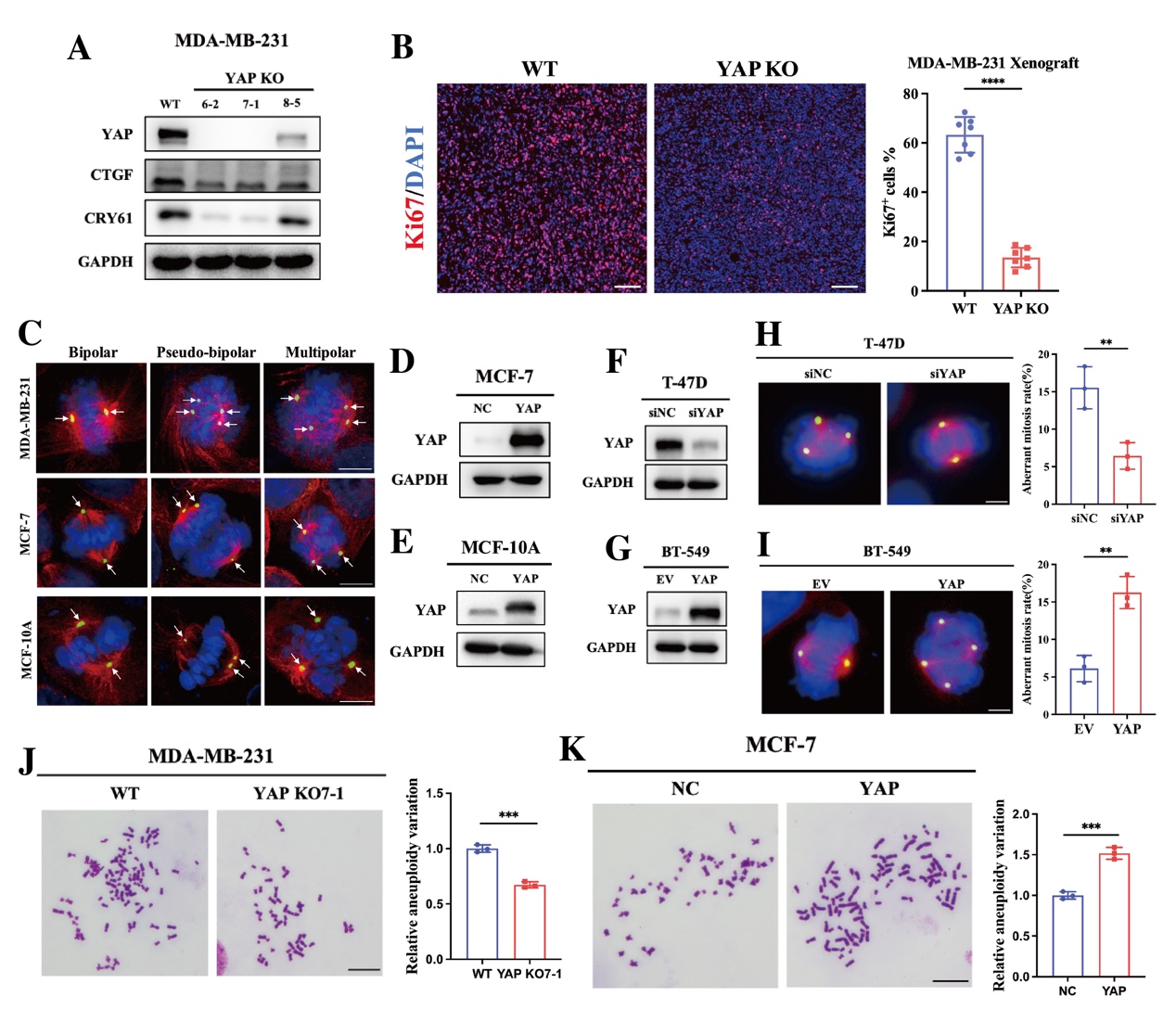
**

**A** Lysates from MDA-MB-231 wild-type (WT) and three YAP-knockout clones (KO6-2, KO7-1, KO8-5) cells were used for immunoblot and probed for YAP, CTGF, and CYR61. GAPDH was used as a loading control.

**B** Immunofluorescence in MDA-MB-231 WT and YAP KO xenograft tumors, and representative images were shown. Ki67 was stained by Cy3 (red), and Nuclei were stained with DAPI (blue), scale bar: 50 μm. Ki67^+^ cells were presented as mean percentage ± SD in histograms. *****p*<0.001.

**C** Representative images of bipolar, pseudo-bipolar, and multipolar mitosis were observed by immunofluorescence in indicated stable MDA-MB-231 (WT and YAP KO7-1), MCF-7 (NC and YAP), as well as MCF-10A (NC and YAP) cells, respectively. β-Tubulin was stained with Alexa Fluor 555 (red), γ-Tubulin was stained with Dylight 488 (green), and Nuclei were stained with DAPI (blue), scale bar: 10 μm.

**D-E** Lysates from MCF-7 (**D**) or MCF-10A (**E**) stable cells with negative control (NC) or YAP overexpression (YAP) were used for immunoblot. Lysates were probed for YAP, and GAPDH was used as a loading control.

**F** T-47D cells with scramble siRNA (siNC) and *YAP* siRNA (siYAP) transfected were collected for immunoblot. Lysates were probed for YAP, and GAPDH was used as a loading control.

**G** BT-549 cells with empty vector (EV) and YAP overexpressing plasmid (YAP) transfected were collected for immunoblot. Lysates were probed for YAP, and GAPDH was used as a loading control.

**H-I** Aberrant mitosis was observed by immunofluorescence in the indicated T-47D (**H**) and BT-549 (**I**) cells. β-Tubulin was stained with Alexa Fluor 555 (red), γ-Tubulin was stained with Dylight 488 (green), and Nuclei were stained with DAPI (blue). Representative images were shown and scale bar: 10 μm. For each experiment, 60-80 mitotic cells were counted, and three independent experiments were performed. Histograms showed the mean percentage ± SD of aberrant mitosis rate. ***p*<0.01. **H** T-47D cells with scramble siRNA (siNC) and *YAP* siRNA (siYAP) transfected. **I** BT-549 cells with empty vector (EV) and YAP overexpressing plasmid (YAP) transfected.

**J-K** Representative images of chromosome metaphase spreading from stable MDA-MB-231 (WT and YAP KO7-1) or MCF-7 (NC and YAP) cells, scale bar: 5 μm. For each experiment, 100-120 cells were counted to quantify the relative aneuploidy variation, and experiments were repeated in triplicates. Data were presented as mean percentage ± SD in histograms. ****p*<0.001.

**Fig S2. YAP/TAZ-TEAD regulates aberrant mitosis in breast cancer.**

**
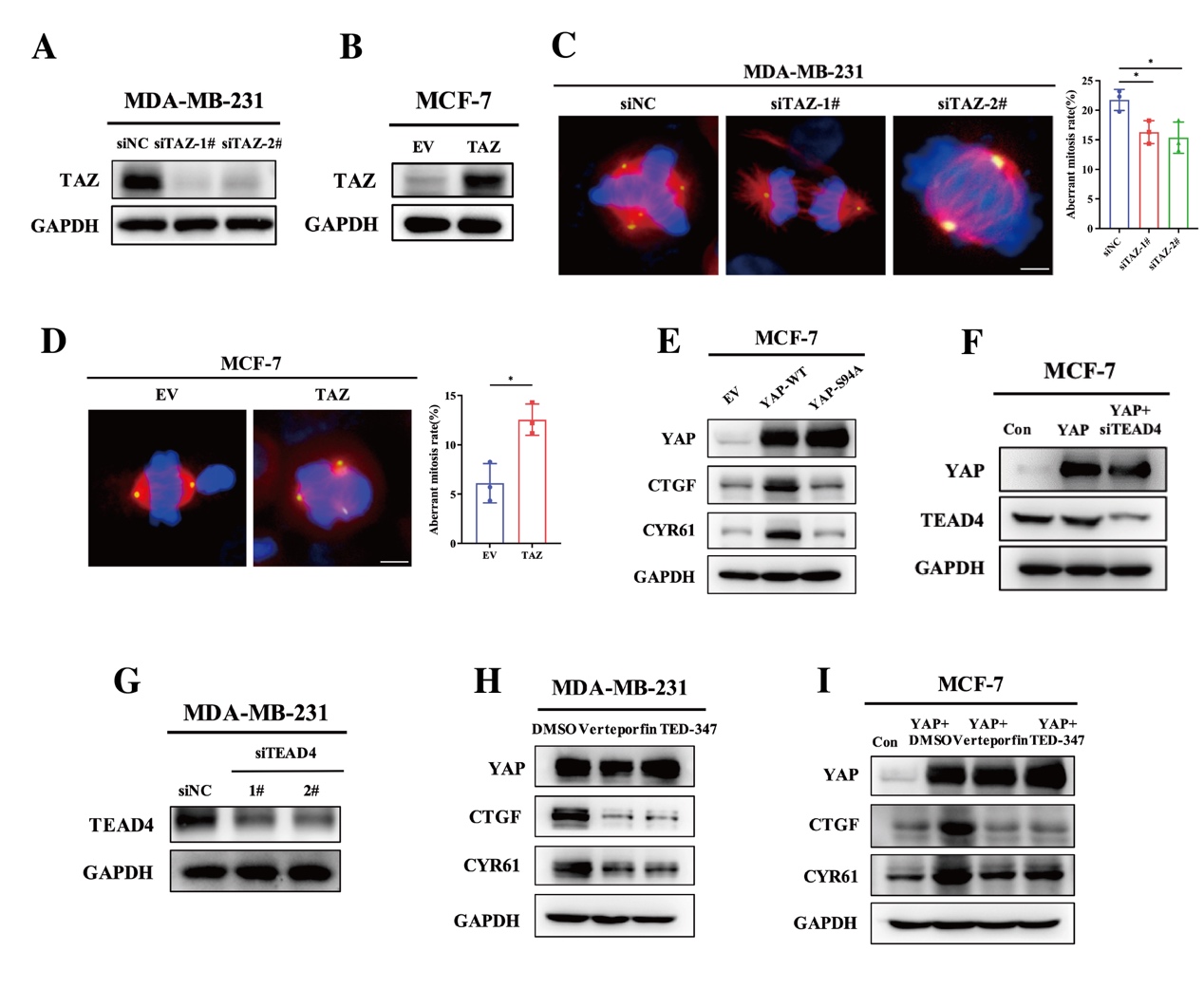
**

**A** Cell lysates from MDA-MB-231 cells transfected with scramble (siNC) or *TAZ* interfering siRNA (siTAZ-1# and siTAZ-2#) were used for immunoblot. Lysates were probed for TAZ, and GAPDH was used as a loading control.

**B** Cell lysates from MCF-7 transfected with empty vector (EV) or TAZ overexpression (TAZ) plasmid were used for immunoblot. Lysates were probed for TAZ, and GAPDH was used as a loading control.

**C-D** Aberrant mitosis was observed by immunofluorescence from MDA-MB-231 (**A**) and MCF-7 (**B**) cells treated as indicated. β-Tubulin was stained with Alexa Fluor 555 (red), γ-Tubulin was stained with Dylight 488 (green), and Nuclei were stained with DAPI (blue). Representative images were shown and scale bar: 10 μm. For each experiment, 60-80 mitotic cells were counted, and three independent experiments were performed. Histograms showed the mean percentage ± SD of aberrant mitosis rate. **p*<0.05.

**E** MCF-7 cells transfected with empty vector (EV), wild-type YAP (YAP WT), or YAP-S94A mutant (YAP S94A) plasmid were used for immunoblot. Cell lysates were probed for YAP, CYR61, and CTGF. GAPDH was used as a loading control.

**F** MCF-7 cells transfected with wild-type YAP plasmid and/or *TEAD4* siRNA (siTEAD4) were used for immunoblot. Cell lysates were probed for YAP and TEAD4. Empty vector and scramble siRNA were used as negative control, and GAPDH was used as a loading control.

**G** MDA-MB-231 cells transfected with scramble (siNC) or *TEAD4* siRNAs (siTEAD4-1#, siTEAD4-2#) were used for immunoblot. Cell lysates were probed for TEAD4. GAPDH was used as a loading control.

**H** MDA-MB-231 cells were treated with DMSO, Verteporfin (1 μM), or TED-347 (10 μM) for 24 h. Western blot assay was performed to examine the protein expression level of YAP, CYR61, and CTGF. GAPDH was used as a loading control.

**I** MCF-7 YAP overexpression (YAP) and control (Con) stable cells were treated with DMSO, Verteporfin (1 μM), or TED-347 (10 μM) for 24 h. Western blot assay was performed to examine the protein expression level of YAP, CYR61, and CTGF. GAPDH was used as a loading control.

**Fig S3. YAP regulates aberrant mitosis through activating AURKA.**

**
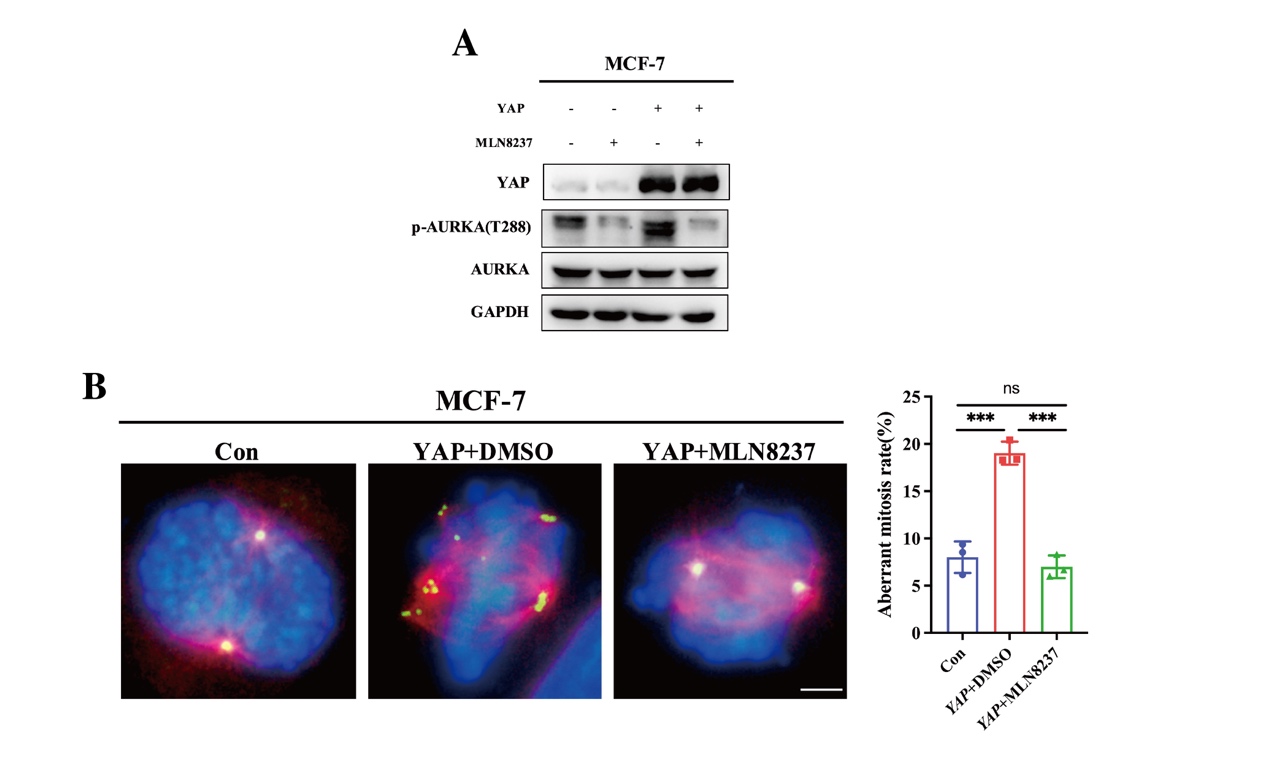
**

**A** MCF-7 YAP overexpression (YAP) and negative control (Con) stable cells were treated with DMSO or MLN8237 at a dose of 10 μM for 24 h, and then were collected for immunoblot. Cell lysates were probed for YAP, AURKA, and p-AURKA (T288). GAPDH was used as a loading control.

**B** MCF-7 control (Con) and YAP overexpression (YAP) stable cells were treated with DMSO or MLN8237 at a dose of 10 μM for 16 hours. Aberrant mitosis was observed by immunofluorescence. β-Tubulin was stained with Alexa Fluor 555 (red), γ-Tubulin was stained with Dylight 488 (green), and Nuclei were stained with DAPI (blue). Representative images were shown and scale bar: 10 μm. For each experiment, 60-80 mitotic cells were counted, and three independent experiments were performed. Histograms showed the mean percentage ± SD of aberrant mitosis rate. ns, not statistically significant; ****p*<0.001.

**Fig S4. YAP/TAZ-TEAD activates AURKA by promoting *AJUBA* transcription.**

**
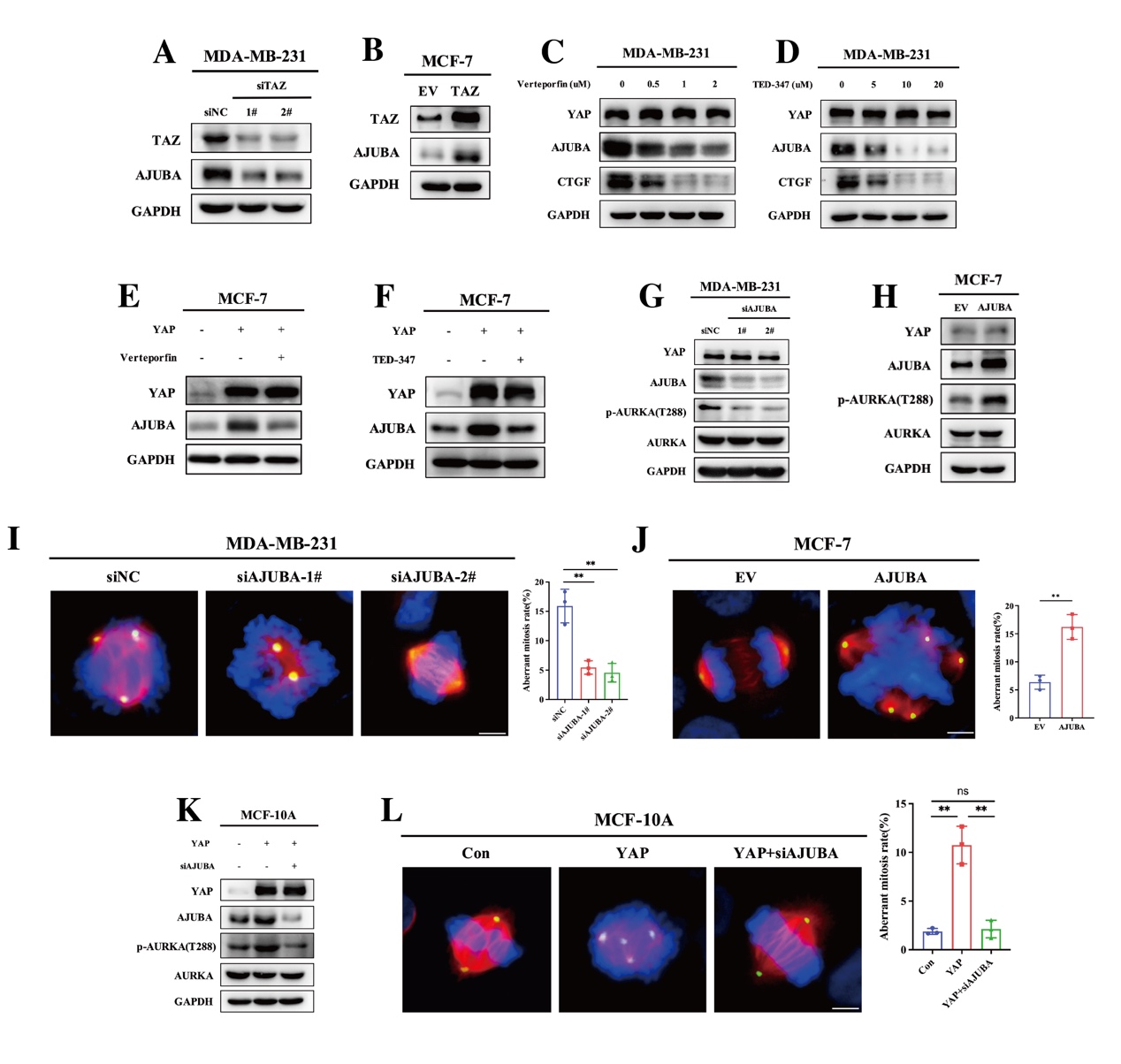
**

**A** MDA-MB-231 cells were transfected with scramble (siNC) or *TAZ* siRNAs (siTAZ-1#, siTAZ-2#). Cell lysates were used for immunoblotting and probed for TAZ and AJUBA. GAPDH was used as a loading control.

**B** MCF-7 cells were transfected with empty vector (EV) or TAZ overexpressing plasmid (TAZ). Cell lysates were used for immunoblotting and probed for TAZ and AJUBA. GAPDH was used as a loading control.

**C-D** MDA-MB-231 cells were treated with DMSO or indicated concentration of Verteporfin (**C**) or TED-347 (**D**) for 24 h. Cell lysates were collected for immunoblot and probed for YAP, AJUBA, and CTGF. GAPDH was used as a loading control.

**E-F** YAP plasmid was transfected into MCF-7 cells. Then, cells were treated with Verteporfin at a dose of 1 μM (**E**) or TED-347 at a dose of 10 μM (**F**) for 24 h. DMSO and empty vector were used as negative control. Cell lysates were collected for immunoblot and probed for YAP and AJUBA. GAPDH was used as a loading control.

**G** MDA-MB-231 cells were transfected with scramble (siNC) or *AJUBA* siRNAs (siAJUBA-1#, siAJUBA-2#). Cell lysates were used for immunoblotting and probed for YAP, AJUBA, AURKA, and p-AURKA (T288). GAPDH was used as a loading control.

**H** MCF-7 cells were transfected with empty vector (EV) or AJUBA overexpressing plasmid (AJUBA). Cell lysates were used for immunoblot, and probed for YAP, AJUBA, AURKA, and p-AURKA (T288). GAPDH was used as a loading control.

**I-J** Representative images of aberrant mitosis in MDA-MB-231 cells transfected with *AJUBA* siRNAs (**I**) or MCF7 cells transfected with AJUBA overexpressing plasmid (**J**). β-Tubulin was stained with Alexa Fluor 555 (red), γ-Tubulin was stained with Dylight 488 (green), and Nuclei were stained with DAPI (blue). For each experiment, 60-80 mitotic cells were counted, and three independent experiments were performed. Histograms showed the mean percentage ± SD of aberrant mitosis rate. Scale bar: 10 μm. ***p*<0.01.

**K** MCF-10A cells were transfected with YAP overexpressing plasmid (YAP) and/or *AJUBA* siRNAs (siAJUBA). Empty vector and scramble siRNA were used as negative control. Cell lysates were probed for YAP, AJUBA, AURKA, and p-AURKA (T288). GAPDH was used as a loading control.

**L** Representative images of aberrant mitosis in MCF-10A cells transfected with YAP overexpressing plasmid and/or *AJUBA* siRNA. β-Tubulin was stained with Alexa Fluor 555 (red), γ-Tubulin was stained with Dylight 488 (green), and Nuclei were stained with DAPI (blue). For each experiment, 60-80 mitotic cells were counted, and three independent experiments were performed. Histograms showed the mean percentage ± SD of aberrant mitosis rate. Scale bar: 10 μm. ns, not statistically significant; ***p*<0.01.

**Fig S5. YAP promotes *AJUBA* transcription through its super enhancer.**


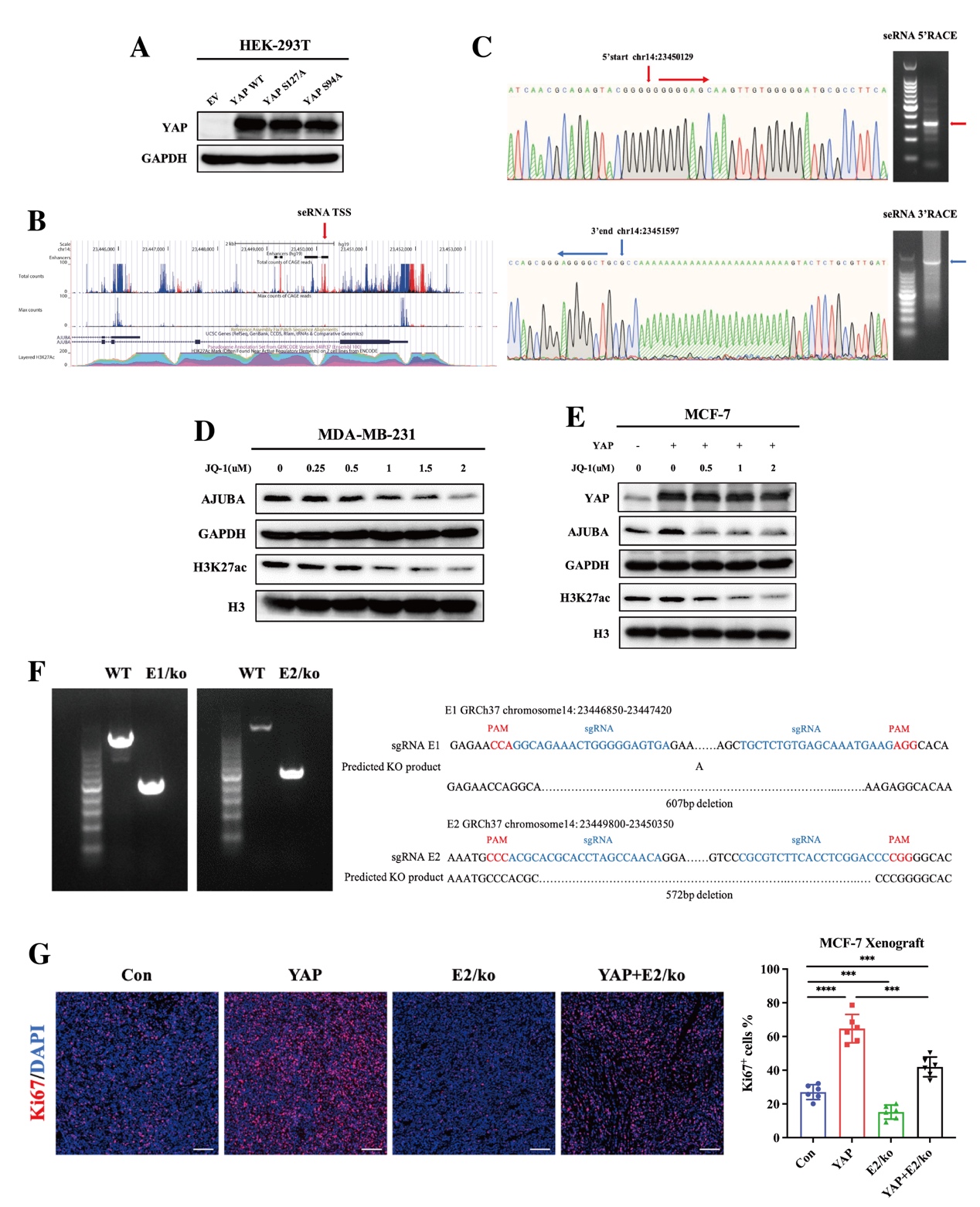


**A** Cell lysates from HEK-293T cells transfected with empty vector (EV), wild-type YAP (YAP WT), YAP-S127A mutant (YAP S127A), and YAP-S94A mutant (YAP S94A) were collected for immunoblot. Lysates were probed for YAP, and GAPDH was used as a loading control.

**B** Diagram of predicted *AJUBA* seRNA transcriptional start site (TSS) according to the FANTOM5 database.

**C** RACE assay and Sanger sequencing analysis were used to validate the *AJUBA* seRNA sequence. The 5' start and 3' end of *AJUBA* seRNA were pointed out by the red arrow and blue arrow, respectively.

**D** MDA-MB-231 cells were treated with the indicated concentration of JQ-1 for 24 h, then the protein levels of AJUBA and H3K27ac were examined via western blot. GAPDH and H3 were used as loading control.

**E** MCF-7 cells transfected with YAP overexpression plasmid (YAP) were treated with the indicated concentration of JQ-1 for 24 h. DMSO and empty vector were used as negative control. The protein levels of YAP, AJUBA, and H3K27ac were examined via western blot. GAPDH and H3 were used as loading control.

**F** PCR amplification and agarose gel electrophoresis were performed to analyze the extracted genome DNA from MCF-7 wild-type (WT) or E1/E2 knockout (E1/ko and E2/ko) cells (left). Indicated PCR product gels were purified and Sanger sequenced to validate the deleted regions at the *AJUBA* enhancer (right). The PAM region was marked in red, and the sgRNA targeted was marked in blue.

**G** Immunofluorescence in MCF-7 Con, YAP, E2/ko, and YAP+E2/ko xenograft tumors and representative images were shown. Ki67 was stained by Cy3 (red), and Nuclei were stained with DAPI (blue), scale bar: 50 μm. Ki67^+^ cells were presented as mean percentage ± SD in histograms. ****p*<0.001; ****p<0.001.

**Supplementary Table**

**Table S1. Key resources table.**

| **REAGENT or RESOURCE** | **SOURCE** | **IDENTIFIER** |
| --- | --- | --- |
| ***Antibodies*** | | |
| Rabbit anti-YAP | Cell signaling technology | Cat. #14074 |
| Rabbit anti-TAZ | Cell signaling technology | Cat. #72804 |
| Rabbit anti-CTGF | Cell signaling technology | Cat. #86641 |
| Rabbit anti-CYR61 | Cell signaling technology | Cat. #14479 |
| Rabbit anti-AJUBA | Cell signaling technology | Cat. #34648 |
| Rabbit anti-AURKA | Cell signaling technology | Cat. #91590 |
| Rabbit anti-p-AURKA(T288) | Cell signaling technology | Cat. #3079 |
| Rabbit anti-BRD4 | Cell signaling technology | Cat. #54615 |
| Rabbit anti-EP300 | Cell signaling technology | Cat. #86377 |
| Rabbit anti-POLR2A | Cell signaling technology | Cat. #14958 |
| Rabbit anti-H3K27ac | Cell signaling technology | Cat. #8173 |
| Rabbit anti-Histone H3 | Cell signaling technology | Cat. #4499 |
| Rabbit anti-Ki-67 | Cell signaling technology | Cat. #9129 |
| Alexa Fluor 555, Rabbit anti-β-Tubulin | Cell signaling technology | Cat. #2116 |
| Rabbit anti-AJUBA | Abcam | Cat. #ab244285 |
| Rabbit anti-TEAD4 | Abclonal | Cat. #A23774 |
| Rabbit anti-γ-Tubulin | Abclonal | Cat. #A9657 |
| Rabbit anti-GAPDH | Abclonal | Cat. #A19056 |
| HRP, Goat Anti-Rabbit IgG | Abbkine | Cat. #A21020 |
| Dylight 488, Goat Anti-Rabbit IgG | Abbkine | Cat. #A23220 |
| Cy3, Goat Anti-Rabbit IgG | Abbkine | Cat. #A22220 |
| ***Bacterial and Virus Strains*** | | |
| DH5α Chemically Competent Cell | Tsingke | Cat. #TSC-C01 |
| ***Biological Samples*** | | |
| human breast cancer tissue arrays | Wuhan Baiqiandu Biotech Company | Cat. #BRC1601 |
| Human multiple tumor tissue array | Xian Alenabio Biotech Company | Cat. #BC000119a |
| ***Chemicals, Peptides, and Recombinant Proteins*** | | |
| TurboFect Transfection Reagent | Thermo Fisher Scientific | Cat. #R0531 |
| Streptavidin T1 Dynabeads | Thermo Fisher Scientific | Cat. #65601 |
| Lipofectamine 2000 | Invitrogen | Cat. #11668019 |
| Lipofectamine 3000 | Invitrogen | Cat. #L3000015 |
| Verteporfin | MCE | Cat. #HY-B0146 |
| TED-347 | MCE | Cat. #HY-125269 |
| MLN 8237 | MCE | Cat. #HY-10971 |
| G418 | MCE | Cat. #HY-K1056 |
| Puromycin dihydrochloride | MCE | Cat. #HY-B1743A |
| Colchicine | Beyotime | Cat. #ST1173 |
| DAPI | Beyotime | Cat. #C1005 |
| Hoechst 33342 | Beyotime | Cat. #C1025 |
| Antifade Mounting Medium | Beyotime | Cat. #P0126 |
| DMSO | PanReac AppliChem | Cat. #A3672 |
| 1,6-Hexaneiol | Macklin | Cat. #H810887 |
| TRIzol | Takara | Cat. #9108 |
| Matrigel | Corning | Cat. #354248 |
| Recombinant Human Insulin | Procell | Cat. #PB180432 |
| ***Critical Commercial Assays*** | | |
| Dual-Luciferase Reporter Assay Kit | Promega | Cat. #E1910 |
| Giemsa Staining Kit | Leagene | Cat. #DM0012 |
| Immunohistochemistry Kit for Rabbit Primary Antibody | Yeasen | Cat. #36312ES50 |
| Fluorescent In Situ Hybridization Kit | RiboBio | Cat. #C10910 |
| mirVana™ miRNA Isolation Kit | Ambion | Cat. #AM1561 |
| TruSeq Stranded mRNA LT Sample Prep Kit | Illumina | Cat. #20020594 |
| MultiS Fast Mutagenesis Kit | Vazyme | Cat. #C215 |
| HiScript-TS 5'/3' RACE Kit | Vazyme | Cat. #RA101-01 |
| Pure Gel DNA Extraction Mini Kit | Vazyme | Cat. #DC301-01 |
| Phanta Max Super-Fidelity DNA Polymerase | Vazyme | Cat. #P505-d1 |
| RT reagent Kit with gDNA Eraser | Takara | Cat. #RR047A |
| TB Green Premix Ex Taq II FAST qPCR Kit | Takara | Cat. #CN830A |
| Protease Inhibitor Cocktail | MCE | Cat. #HY-K0010 |
| Phosphatase Inhibitor Cocktail I | MCE | Cat. #HY-K0021 |
| Phosphatase Inhibitor Cocktail II | MCE | Cat. #HY-K0022 |
| West Pico PLUS Chemiluminescent Substrate Kit | Thermo Fisher Scientific | Cat. #34580 |
| BCA Protein Assay Kit | Thermo Fisher Scientific | Cat. #23225 |
| Universal Virus Concentration Kit | Beyotime | Cat. #C2901 |
| Hematoxylin and Eosin Staining Kit | Beyotime | Cat. #C0105S |
| Simple ChIP Enzymatic Chromatin IP Kit | Cell signaling technology | Cat. #9003 |
| FlexAble 2.0 CoraLite® Plus 647 Antibody Labeling Kit for Rabbit IgG | Proteintech | Cat. #KFA503 |
| ***Deposited Data*** | | |
| H3K4me1 ChIP-seq on human MCF-7 | ENCODE | ENCSR493NBY |
| H3K4me3 ChIP-seq on human MCF-7 | ENCODE | ENCSR000DWJ |
| H3K27ac ChIP-seq on human MCF-7 | ENCODE | ENCSR000EWR |
| CTCF ChIP-seq on human MCF-7 | ENCODE | ENCSR000DML |
| POLR2A ChIP-seq on human MCF-7 | ENCODE | ENCSR000DMT |
| TEAD4 ChIP-seq on human MCF-7 | ENCODE | ENCSR000BUO |
| MCF-7 intact Hi-C | ENCODE | ENCSR660LPJ |
| YAP ChIP-seq on MDA-MB-231 | GEO database | GSE66081 |
| TEAD4 ChIP-seq on MDA-MB-231 | GEO database | GSE66081 |
| Expression profiling of siYAP/TAZ on MDA-MB-231 | GEO database | GSE66082 |
| ***Experimental Models: Cell Lines*** | | |
| MCF-7 | ATCC | ATCC HTB-22 |
| MDA-MB-231 | ATCC | ATCC HTB-26 |
| MCF-10A | ATCC | ATCC CRL-10317 |
| HEK-293T | ATCC | ATCC CRL-1573 |
| T-47D | ATCC | ATCC HTB-133 |
| BT-549 | ATCC | ATCC HTB-122 |
| ***Experimental Models: Organisms/Strains*** | | |
| BALB/c nude female mice | Gempharmatech Co., Ltd | N/A |
| ***Oligonucleotides*** | | |
| sgRNAs for CRISPR/Cas9 knockout, dCas9 guide CRISPR a/i and dCas9 guide CAPTURE system, see Table S2 | This paper | N/A |
| Primers for site-directed mutagenesis, see Table S3 | This paper | N/A |
| siRNAs for transient gene knockdown, see Table S4 | This paper | N/A |
| Primers for RT-qPCR, see Table S5 | This paper | N/A |
| Primers for ChIP, ChIP-qPCR, Enhancer KO validation, and seRNA RACE, see Table S6 | This paper | N/A |
| ***Recombinant DNA*** | | |
| pcDNA3.1-3×Flag -YAP-WT | This paper | N/A |
| pcDNA3.1-3×Flag-YAP-S127A | This paper | N/A |
| pcDNA3.1-3×Flag-YAP-S94A | This paper | N/A |
| pcDNA3.1-V5-YAP-WT | This paper | N/A |
| pcDNA3.1-V5-YAP-4LE | This paper | N/A |
| pcDNA3.1-mEGFP-YAP-WT | This paper | N/A |
| pcDNA3.1-mEGFP-YAP-4LE | This paper | N/A |
| pcDNA3.1-mCherry-YAP-WT | This paper | N/A |
| pcDNA3.1-HA-TAZ | This paper | N/A |
| pcDNA3.1-3×Flag-AJUBA | This paper | N/A |
| pLVX-Neo | MiaoLingBio | Cat. #P0247 |
| psPAX2 | Addgene | Cat. #12260 |
| pMD2.G | Addgene | Cat. #12259 |
| pSpCas9(BB)-2A-Puro-sgRNA | Addgene | Cat. #62988 |
| sgRNA (MS2) cloning backbone | Addgene | Cat. #61424 |
| SP-dCas9-VPR | Addgene | Cat. #63798 |
| dCas9-KRAB-MeCP2 | Addgene | Cat. #110821 |
| pLVX-EF1a-BirA-P2A-FB-dCas9-IRES | Addgene | Cat. #138417 |
| mEGFP-dCAS9 | This paper | N/A |
| PGL3-Basic | Promega | Cat. #E1751 |
| pGL3-Enhancer | Promega | Cat. #E1771 |
| pRL-TK | Promega | Cat. #E2241 |
| ***Software and Algorithms*** | | |
| SPSS (version 22.0) | IBM | N/A |
| GraphPad Prism (version 9.5.0) | GraphPad | N/A |
| ImageJ (version 1.5.4h) | NIH | N/A |
| R software (version 4.3.2) | R Project | N/A |
| Bowtie2 (version 2.5.2) | http://bowtie-bio.sourceforge.net | N/A |
| Bedtools (version 2.31.0) | https://bedtools.readthedocs.io | N/A |
| IGV (version 2.16.2) | https://igv.org | N/A |
| HiC-Pro pipeline (version 3.1.0) | https://github.com/nservant/HiC-Pro | N/A |
| ChIPseeker (version 3.18) | https://bioconductor.org/packages/  release/bioc/html/ChIPseeker.html | N/A |
| ROSE Software | https://bitbucket.org/young_computation/rose/src/master/ | N/A |
| DAVID | https://david.ncifcrf.gov/tools.jsp | N/A |
| JASPAR | https://jaspar.elixir.no/ | N/A |
| ***Others*** | | |
| MEGM Kit | Lonza | Cat. #CC-3150 |
| Leibovitz's L-15 Medium | Gibico | Cat. #11415064 |
| DMEM Medium | Gibico | Cat. #11995065 |
| RPMI-1640 Medium | Gibico | Cat. #11875168 |
| Opti-MEM I Reduced Serum Medium | Gibco | Cat. #31985070 |
| Fetal Bovine Serum | Gibco | Cat. #10091148 |

**Table S2. sgRNAs for CRISPR/Cas9 knockout, dCas9 guide CRISPR a/i and dCas9 guided CAPTURE system.**

| **Name** | **Target Sequence (5`-3`)** |
| --- | --- |
| ***sgRNAs for YAP KO*** | |
| sgRNA-*YAP*-1# | GCAGTCGCATCTGTTGCTGC |
| sgRNA-*YAP*-2# | GAGCACTCTGACTGATTCTC |
| sgRNA-*YAP*-3# | ACATCGATCAGACAACAACA |
| ***sgRNAs for E1/E2 KO*** | |
| E1KO-1# | TCACTCCCCCAGTTTCTGCCTGG |
| E1KO-2# | TGCTCTGTGAGCAAATGAAGAGG |
| E2KO-1# | TGTTGGCTAGGTGCGTGCGTGGG |
| E2KO-2# | CGCGTCTTCACCTCGGACCCCGG |
| ***sgRNAs for CRISPR a/i*** | |
| sgRNA-E1-1# | GTGAAAGGGGGGAGCAAGTT |
| sgRNA-E1-2# | GAATTCGGAGAATGCGCGGG |
| sgRNA-E2-1# | GTGCGCCTTCACTGCCCCACT |
| sgRNA-E2-2# | GCGGCGCGCACAAACCTCCA |
| ***sgRNAs for dCas9 guided CAPTURE*** | |
| sgRNAs-E1 | E1-1: GTTTAGCAAAGGAATCAGAA  E1-2: GCAGTAAGGAGGGGGTAATG  E1-3: GGGGGGCAGTTAAGGCCGCT  E1-4: GAGCATCCCCCCTTTCCAAG  E1-5: GGAGCGTGATGTCATCAGCT  E1-6: GCTGTTTGGGGCGGAGAGGG  E1-7: GGAGGGTGGGGGCAGGCTGG  E1-8: GACAGTGAAAGACTGGAATG  E1-9: AGCCCCACCAAGAAGCTGGA  E1-10: AAGTCCAGGATGGGCAAGCA  E1-11: GGGAGAAACAGAGGTGAGGA  E1-12: AAAGGGGACCCACCAGGCGG |
| sgRNAs-E2 | E2-1: GAGACAGGATGGAGCGCCCT  E2-2: GCGGAGTAGTGGCGGCTGTC  E2-3: GGGTGGGGTTCGGGGATCGG  E2-4: GTGCGTGCCGACTCCTGCCC  E2-5: GAATTCCTGCGGGCGGAGCC  E2-6: GTGCGCGCCGCGCCCCCACC  E2-7: GGGGCACCGCCGTGAAAGGG  E2-8: TGCGCCTTCACTGCCCCACT  E2-9: GCAGGAGGCCTCGTAGGGAA  E2-10: GGACTCACGAGATACCAGGG  E2-11: GCGGCGCGCACAAACCTCCA  E2-12: TTAGCAGAAATAAGGAGCCC |

**Table S3. Primers for site-directed mutagenesis.**

| **Name** | **Forward** | **Reverse** |
| --- | --- | --- |
| E1-MUT | CCTCCCCACCGCACAGTCTTTCACTGTCGCCCCAGGC | TGAAAGACTGTGCGGTGGGGAGGGTGGGGGCAGGCT |
| E2-MUT | CATTCTCCGACGCACTGCGGGCGGAGCCTGGGCCCGGG | CCGCCCGCAGTGCGTCGGAGAATGCGCGGGTGGGGG |
| Promoter-MUT | GGCATGGTGTGCGGTGTCGCGTCCCGGGACCGGACG | GACGCGACACCGCACACCATGCCCCTCCGCGCATTCCAA |
| YAP-4LE-MUT-1 | cgagagcagcaagagcagATGGAGAAGGAGAGGCTGCG | ctgctcttgctgctctcgCATCTGTTGCTGCTGGTTGGA |
| YAP-4LE-MUT-2 | cggctgaaacagcaagaagagCTTCGGCAGGCAATGCGG | ttcttgctgtttcagccgctcCCTCTCCTTCTCCATCTGCTCTT |

**Table S4. siRNAs for transient gene knockdown.**

| **Name** | **Target Sequence (5`-3`)** |
| --- | --- |
| si*YAP*-1# | GCGTAGCCAGTTACCAACA |
| si*YAP*-2# | GGTGATACTATCAACCAAA |
| si*TAZ*-1# | TGCTTCCTCAGTTACACAAAG |
| si*TAZ*-2# | TCCTAACAGTCCGCCCTACTT |
| si*TEAD4*-1# | GCTTGTGGATGAAGTTGAT |
| si*TEAD4*-2# | GGACACTACTCTTACCGCA |
| si*AJUBA*-1# | TGGACCGGGATTATCACTTTG |
| si*AJUBA*-2# | GTGTCTGTGGTCACTTGATTT |

**Table S5. Primers for RT-qPCR.**

| **Name** | **Forward** | **Reverse** |
| --- | --- | --- |
| *YAP* | TAGCCCTGCGTAGCCAGTTA | TCATGCTTAGTCCACTGTCTGT |
| *TAZ* | GATCCTGCCGGAGTCTTTCTT | CACGTCGTAGGACTGCTGG |
| *CTGF* | AAAAGTGCATCCGTACTCCCA | CCGTCGGTACATACTCCACAG |
| *CRY61* | CTCGCCTTAGTCGTCACCC | CGCCGAAGTTGCATTCCAG |
| *AJUBA* | ATGGGGAAGTCCTATCATCCAG | TGGTAGTCGGTGACACAGTAT |
| *seAJUBA* | ACTTGGTCCCTTCCCTACGA | ACTGTCCCCTTCCTGCTAGT |
| *SNAI1e* | GTGTGTTCTGGTCTGTTCTAGAACC | GGAAGACTGCCTGGCACACA |
| *GAPDH* | GGAGCGAGATCCCTCCAAAAT | GGCTGTTGTCATACTTCTCATGG |

**Table S6. Primers for ChIP, ChIP-qPCR, Enhancer KO validation, and seRNA RACE.**

| ***Primers for CHIP and CHIP-qPCR*** | | |
| --- | --- | --- |
| **Name** | **Forward** | **Reverse** |
| E1 | GAGGTGGCTGAACTCTGACC | GGCGACAGTGAAAGACTGGA |
| E2 | ACTTGGTCCCTTCCCTACGA | CCCCCTGGTATCTCGTGAGT |
| Promoter | GCTAGCCACAGACCGAACTT | CAGAACATGCCTCCTGTCGT |
| ***Primers for Enhancer KO validation*** | | |
| **Name** | **Forward** | **Reverse** |
| E1/ko | CCCAggTgTCTACATTATCAgggATAgg | ACACTTTTCTCTTCTCAggCACCT |
| E2/ko | ATCAATACTTCCTgAACAgAgTAggggAg | CgCggAgAACCCTCgg |
| ***Gene specific primers for seRNA RACE*** | | |
| 3' GSP primer | | GCAGGAAGGGGACAGTGCTCTGGGGC |
| 5' GSP primer | | GGGTCCGAGGTGAAGACGCGGG |
